# Effects of Neutral, Anionic and Cationic Polymer Brushes Grafted from Poly(para-phenylene vinylene) and Poly(para-phenylene ethynylene) on the Polymer’s Photoluminescent Properties

**DOI:** 10.3390/polym14142767

**Published:** 2022-07-06

**Authors:** Thomas Kerr-Phillips, Mona Damavandi, Lisa I. Pilkington, Kathryn A. Whitehead, Jadranka Travas-Sejdic, David Barker

**Affiliations:** 1 School of Chemical Sciences, University of Auckland, Private Bag, Auckland 92019, New Zealand; tker016@aucklanduni.ac.nz (T.K.-P.); mdam579@aucklanduni.ac.nz (M.D.); lisa.pilkington@auckland.ac.nz (L.I.P.); j.travas-sejdic@auckland.ac.nz (J.T.-S.); 2 The MacDiarmid Institute for Advanced Materials and Nanotechnology, Victoria University of Wellington, Wellington 6012, New Zealand; 3 Microbiology at Interfaces, Manchester Metropolitan University, Chester Street, Manchester M1 5GD, UK; k.a.whitehead@mmu.ac.uk

**Keywords:** molecular engineering, grafted polymers, fluorescence

## Abstract

The conformation of a fluorescent polymer, in the solid state or in solution, plays a critical role in the polymer’s fluorescent properties. Thus, grafted side chains on a fluorescent polymer can directly influence its optical properties. In this study, the effect of grafted polymeric side chains on the photoluminescent properties of poly(para-phenylene vinylene) (PPV) and poly(para-phenylene ethynylene) (PPE) were investigated. Low- and high-molecular-weight grafts of neutral poly(n-butyl acrylate), cationic poly(trimethylaminoethyl methacrylate) and anionic poly(sulfopropyl acrylate) were grafted onto PPVs and PPEs, and the effect of the grafting on the graft copolymer’s absorption and emission wavelengths, the fluorescence intensity and the quantum yield were studied. The results indicate that in the case of the ionic grafts, contrary to the expectations, the polymers have a reduced quantum yield. This contrasts with the copolymers with uncharged side chains (P*n*BA), where a major increase in the quantum yield is seen for the self-quenching conjugated pristine polymers. These results reinforce that the molecular conformation of the polymer in a solid or solution plays a critical role in fluorescent polymers photoluminescent properties.

## 1. Introduction

It is well known that a copolymer’s molecular structure directly affects its physical properties; random copolymers tend to have averaged properties of the individual monomers, block polymers tend to show the properties of each of the corresponding blocks and graft copolymers generally have the properties of the pristine grafted polymer [1,2]. Thus, with molecular engineering, the properties of materials can be carefully tuned to a desired application. This applies to physical properties such as glass transition, elastic modulus and solubility, but the structure–property relationships are more complicated when it comes to other properties such as electronic and optical properties [3]. There are cases where the bulk physical properties can be tailored through macromolecular design, while maintaining the electronic or optical properties [4,5,6]. In conjugated copolymer systems, the copolymers’ inter- and intramolecular interactions play a large role in the optical and electronic properties. Thus, simple molecular design considerations may not be enough to predict the resulting materials properties, where the optical and electronic properties are dictated also by copolymer conformation that can be affected by even minute changes in the environmental conditions, such as temperature or solvent quality.

Some conjugated polymers (CPs) have strong fluorescent properties and have been shown to be a useful material in optical sensing technologies [7,8,9], light emitting diodes [10] and solar cells [11,12]. Over the last two decades, research has emphasized how important the molecular conformation and molecule–molecule spacing and interactions are for the device’s optical properties [13,14,15]. Self-quenching is one of the consequences of such inter/intra molecular interactions, where non-radiative decay occurs through hole hoping, internal conversion of the excited states and vibrational relaxation [16]. This often results in poor quantum yields, both in the solid state and in solution. Recently, we have shown that the quantum yield can be drastically increased for PPV’s by the addition of neutral poly(butyl acrylate) grafts [5]. This was attributed to the effect of physical spacing of the individual chromophores by the large grafts preventing intermolecular interactions of the polymer backbone, thus preventing self-quenching. While the grafting process caused a blue shift in the fluorescence, due to the addition of defects on the polymer backbone, the quantum yield increased (from 42 to 87%). The polymer’s physical properties were also modified by the addition of P*n*BA grafts. The grafted PPVs become more soluble in common organic solvents, and therefore much more processable, and showed a lower glass transition temperature than pristine PPV. Based on these results, it was decided to investigate whether the same effects would translate to ionic grafts in aqueous solutions.

There are many applications for anionic [17,18,19,20,21] and cationic [22,23,24] fluorescent polymers. Cationic fluorescent polymers, specifically those of the quaternary ammonium [25] variety, have strong antimicrobial properties and anion sensing abilities [26], whereas anionic fluorescent polymers have been shown to be useful for cation sensors [27,28] and biosensors [29].

In this study, the effects of grafting ionic side chains (poly (3-(acryloyloxy)propane-1-sulfonate), PSPA and poly 2-(methacryloyloxy)ethyl]trimethylammonium chloride, PMETAC) on PPV and PPE backbones on the graft-copolymers’ optical properties were studied and compared against that of neutral grafts.

## 2. Materials and Methods

### 2.1. Materials 

All chemicals were purchased from Sigma Aldrich or AK Scientific and used without purification.

### 2.2. Synthesis 

All air and water-sensitive synthetic manipulations were performed under nitrogen atmosphere using standard techniques. For complete synthesis details please see the Supporting Information. PPV and PPE monomer and macroinitiator synthesis was performed as previously reported [26,30].

### 2.3. General Atom Transfer Radical Polymerization Method

Atom Transfer Radical Polymerization (ATRP) was conducted using the Activators ReGenerated by Electron Transfer (ARGET) method as follows: The polymer macroiniator was dissolved in an appropriate solvent (DMF or DMSO); to this, an internal standard (anisole) was added in stoichiometric amounts, which was followed by the monomer (and, if necessary, small amounts of water as a cosolvent), and the resulting solution was degassed through freeze–thaw cycles. To this solution, the Cu(II) complex with ligand, tri(2-pyridylmethyl)amine (TPMA) or (N,N,N′,N″,N″-pentamethyldiethylenetriamine) (PMDTA) in a small amount of solvent was added and the solution was heated to 60 or 65 °C. The reducing agent (tin(II) ethyl hexanoate or ascorbic acid) was added and the reaction was left to run for varying times. The general system ratios were as follows: MI:Monomer:Copper:Ligand 1:1000:1.5:4. Note that due to solubility constraints, some variations of this general procedure was conducted.

^1^H NMR spectra of graft-copolymers were recorded on a 400 MHz Bruker instrument. UV–visible absorption spectra were measured with a UV–Visible spectrophotometer (Pharmaspec UV-1700, Shimadzu). Fluorescence spectra were measured with a Perkin Elmer LS 55 spectrophotometer with a 3-Q-10 mm rectangular quartz cell, at varying excitation wavelengths. Solution-state quantum yields were determined using either PPV or PPE grafted polymer samples in toluene or water relative to anthracene 314 in ethanol (φ = 0.27). Solid-state samples were prepared by drop-casting the relevant polymer solution (5 mg mL^−1^ in toluene) onto a quartz slide and solvent annealed with toluene. The quantum yield was then determined using an integrating sphere [31,32], exciting at 360 nm.

The molecular weights of the grafted polymers were determined by Gel Permeation Chromatography (GPC) using a Viscotek TDAmax from Malvern Instruments equipped with either a Plgel 5 mm Mixed-C (300 × 7.5 mm) column or 2 × A5000 (300 mm × 8 mm each) Viscotech columns and A7Guard (50 mm × 8 mm) Guard column. THF or deionized water (MilliQ, 18.2 MΩ cm) containing 0.02% NaN_3_ was used as the eluent versus polystyrene standards (PolySciences) or dextran standards (Sigma-Aldrich) [26,30]. Typical ^1^H NMR spectra for the backbone and grafted polymers can be seen in Supporting Information, Figures S2–S8. In the cases of grafted copolymers, the NMR spectra is also accompanied with the NMR spectra of the grafted monomer.

## 3. Results and Discussion

### 3.1. Grafting of Poly(cation), Poly(anion) and Poly(n-butly actrylate) from PPVs and PPEs

**Figure 1:**
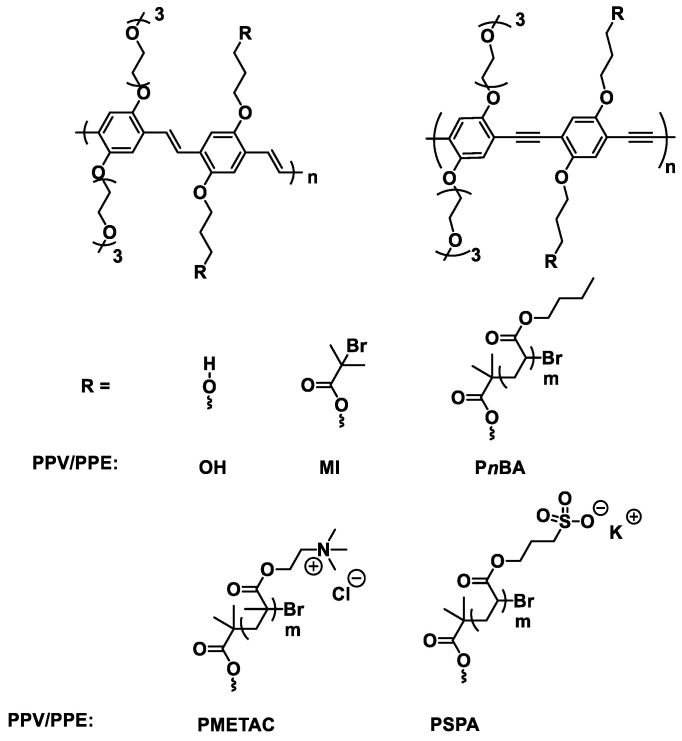
Chemical structures for the PPV and PPE macroinitiators and the resulting polymers.

Within this study, fluorescent, laterally grafted copolymers based on PPV and PPE with poly(*n*-butlyacrylate) (P*n*BA), PSPA and PMETAC as grafts were examined (Figure 1). Similar polymers have been developed by us previously and we have shown that the addition of the triethylene glycol substituent on the PPV and PPE backbone (e.g., **PPVOH**/**PPEOH,** Figure 1) improves the solubility of the grafted PPVs and PPEs in various solvents, which allows easy subsequent functionalization [33]. The alpha-acyl bromide functionality found in macroinitiators (**PPVMI**/**PPEMI**, Figure 1) supports later ATRP-based grafting that can be used to tailor the polymer’s physical properties while still maintaining the backbone polymers’ optical properties.

**Figure 2:**
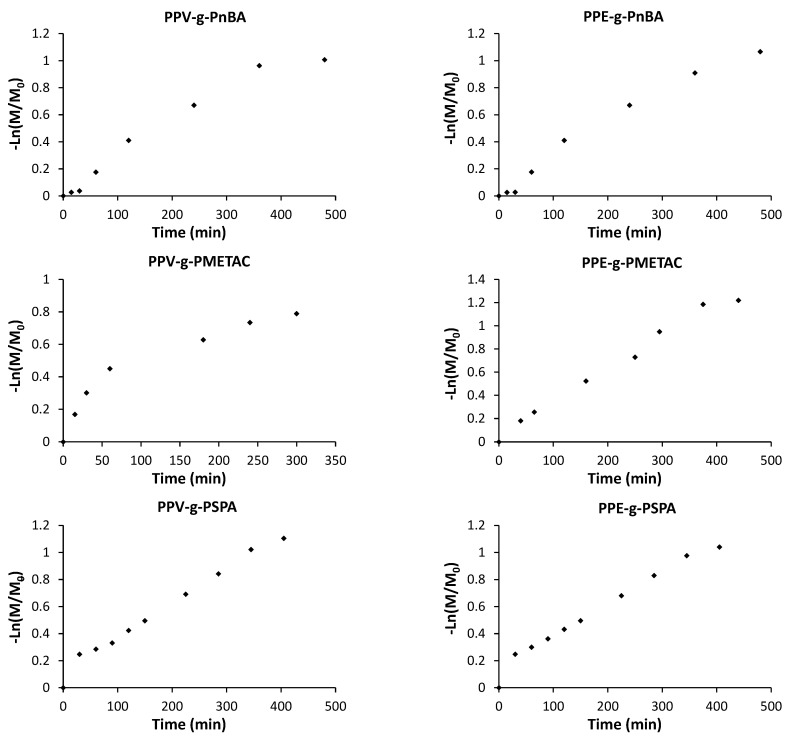
Kinetics of grafting of neutral (P*n*BA), cationic (PMETAC) and anionic (PSPA) grafts from PPVMI and PPEMI macroinitiators by ATRP.

The kinetics of polymerization of the grafts (Figure 1) via ATRP from **PPVMI** and **PPEMI** were monitored by means of NMR with the use of an internal standard (anisole), as published previously [5]. From the results presented in Figure 2, it can be seen that the kinetics diverges from the expected first order kinetics for ATRP, especially at the later times of polymerizations and for the P*n*BA grafted polymers.

The closest to first-order kinetics was grafting polymerization of PSPA from **PPVMI** and **PPEMI** (**PPV-*g*-PSPA, PPE-*g*-PSPA**), which shows only slight deviation from first order (Table 1). For the determination of linearity, the first few data points are ignored for P*n*BA and PSPA grafting as there are determined to be an initiation period (i.e., the time zero point was not included). The ATRP kinetics reveals much about how each of these polymer systems are behaving in their respective solutions during the grafting process. Assuming that termination rates are low and that all other reactant’s concentration remain the same, anything higher than first order implies that the monomer has an additional role in the overall reaction. Commonly, in ATRP, this could be termination reactions, chain transfer reactions or other side reactions, such as radicals reacting with the backbone polymer. We postulate that in this case, in addition to some to the aforementioned causes, the monomer is needed for keeping the polymers in solution by forming a solvation sphere and more monomer is needed as the brushes grow. It must be mentioned that the macroinitiator polymer will have a very different solubility to the grafted one, and thus, initial kinetics would be linear until the polymer’s solubility is sufficiently changed. This could explain what appears to be two separate phases in PMETAC polymers, where at some point, the solubility changes enough to drastically decrease the reaction rate. In the case of both of the P*n*BA grafted polymers, the reaction is second order. As this appears to be a more continual process (shown by the curvature) and ignoring the slow initiation, this is more likely due to chain transfer and only partially due to the proposed solubility changes.

The kinetics results are supported by GPC data and are shown in the Supporting Information, Table S1. As seen in the GPC data, increased grafting time increases the polymers molecular weight. It should be noted that since the GPC was performed using linear polystyrene standards in THF, these results provide only an estimate of the molecular weights, with the molecular weight increasingly overestimated as the polymer is grafted with longer grafts. This is due to the decreased radius of hydration of grafted polymers compared to the linear reference [34]. This is likely why the GPC results overestimates molecular weights for the 24 grafted copolymers to that estimated from NMR experiments (see Supporting Information for details).

The general ATRP grafting method could not be applied directly to grafting of PSPA. SPA is sparingly soluble in DMSO, and the PSPA graft copolymers are even less soluble, with the properties of the poorly soluble PSPA grafts eventually overcoming the inherent solubility of the **PPVMI**/**PPEMI** backbones, causing the grafted polymers with relatively short graft lengths to be insoluble in DMSO. It was thought that using PPV and PPE monomers would improve the overall solubility enough to facilitate successful ATRP. However, these initial attempts to overcome this only further demonstrated the solubility issue, where PPV and PPE monomers (see Supporting Information) were used as ATRP initiators with poor results. In all cases, early termination of the ATRP reaction was seen, due to the polymer precipitating out of the solution. The synthesis of these graft copolymers was overcome by utilizing phase transfer complexation (PTC). It is known that crown ethers can be used to coordinate potassium ions, which results in complexes that can be drastically more soluble in organic solvents [35,36]. Thus, using 18-crown-6, the SPA’s solubility was improved [35,36]. Subsequent ATRP on macroinitiators **PPVMI** and **PPEMI** using the SPA monomer with 18-crown-6 facilitated successful ATRP, forming **PPV-*g*-PSPA** and **PPE-*g*-PSPA**. Furthermore, it is hypothesized that PTC method also improve the solubility of the grafted polymers in the DMSO solvent, thus causing more linear kinetics.

Thus, both the PPV and PPE were successfully laterally grafted with a neutral, anionic and cationic polymer. As expected, the resulting copolymers had physical properties resembling closer the grafts as opposed to the polymer backbone.

### 3.2. Optical Properties of the Grafted Copolymers

#### 3.2.1. PPVs and PPEs Grafted with Uncharged Side Chains

Previously, we have shown that ATRP grafting can alter the optical properties of PPVs in the solid state and in solution [5]. The altered optical properties of the previous study was achieved through lateral grafting of P*n*BA on two separate PPV derivatives and the optical properties had changed in such a way that a blue shift in the absorption and emission spectra upon grafting, as well as a substantial increase in the quantum yields, were observed (Table 2). The increase in quantum yields was also seen for solid-state systems, like seen previously [5], with the PPVs going from 0% for the ungrafted PPVs and PPEs to 15 ± 2 and 21 ± 2 for the grafted (24 h) PPVs and PPEs (24 h), respectively.

**Figure 3:**
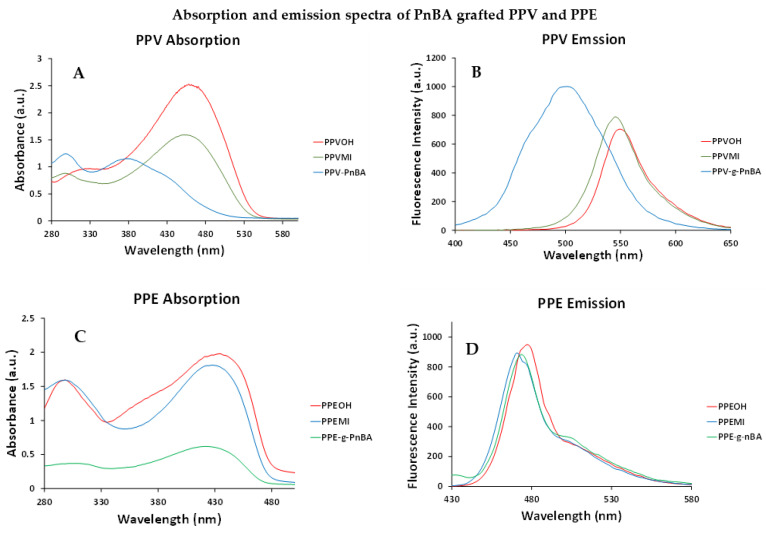
Absorption and emission spectra for **PPVOH**, **PPVMI** and **PPV-*g*-P*n*BA** in DMF (**A**,**B**) and spectra for **PPEOH**, **PPEMI** and **PPE-*g*-P*n*BA** in DMF (**C**,**D**).

In this study, three separate copolymer PPV and two PPE samples were synthesized with increasing P*n*BA graft lengths. Like in the aforementioned study, we saw a substantial blue shift and broadening in the absorption and emission (Figure 3) for all PPV grafted copolymers. This blue shift and broadening is attributed to the addition of parylene type defects in the backbone, which reduces the conjugation length and increases the length distribution of conjugated segments.

When the same grafting process is applied to PPEs (for two different graft lengths controlled by polymerization time), the results differ from that of **PVP-*g*-P*n*BA**. With PPEs, there is only a relatively small blue shift of 7 nm in the absorption (Figure 3, left) between the ungrafted and grafted polymers. Furthermore, there is no discernible shift in the fluorescence (Figure 3, right). This is attributed to PPE’s superior stability towards radicals and higher oxidation potential, resulting in less addition of defects via the formation of parylene type units [37]. There is no discernable increase in the grafted PPE’s quantum yield in solution as the quantum yield was already 87% for PPE in solution. This implies that in DMF, the ungrafted PPEs are not aggregated, and thus, no self-quenching occurs, and this remains the case with the grafted PPEs. The solid-state results are as expected, where an increase in the quantum yield from 0% to 21% is due to the grafted chain physically separating the PPE backbones.

#### 3.2.2. PPVs and PPEs Grafted with Ionic Side Chains

Due to a reduced solubility of the grafted copolymers in organic solvents, the length of ionic grafts was controlled through the concentration of monomer in the reaction mixture. Monomer to macroinitiators ratios was kept at either 500:1 or 250:1 mol:mol and polymersiation time was kept constant (3 h). The blue shift, peak broadening and the appearance of the peak at 300 nm can be used to determine the relative amount of defects added to the conjugated polymers backbone. It can be seen that both of the PPV ionic grafted polymers show that considerable amounts of defects are added during the grafting process, more so than during the grafting of neutral (P*n*BA) grafts. However, the cationic PMETAC UV–Vis spectra indicates a much higher amount of defects are added. This is seen by the large blue shift in the absorption spectra of 107 nm in the PSPA grafted polymers but only 20 nm in the PMETAC grafted polymers, compared to the 73 nm in P*n*BA grafts. The sharp peak in the UV–Vis for **PPV-*g*-PSPA** is attributed to phenylene type units, which are isolated due to defects. Similar peaks at 300 nm are seen for other PPVs [38] and seen on the neutral grafted PPV (Figure 3).

When comparing the fluorescence spectra and quantum yield (Table 3), from the decrease in quantum yield it is clear that large amounts of self-quenching occur for both ionic grafts. This is somewhat contrary to intuition and what is seen in some literature [39,40], as one would expect that charged grafts would repel each other, forcing the molecules to physically separate. There are several possible explanations for this unexpected decrease in quantum yield. Firstly, one explanation is that molecular aggregations similar to micelles are occurring. In this situation, we propose a similar system to that described by Shin et al. [41], and we considered the backbone to be the hydrophobic head of a ‘reverse’ surfactant (reverse surfactant as described by Machinn et al. [42]). Secondly, another explanation is that the large, charged polymeric side chains both prevent planarization [43] and introduce more non-radiative decay pathways through charge transfer, thus decreasing quantum yield. While the prevention of molecular planarization cannot be a major factor as the neutral grafts do not show the same decrease in quantum yield, the charged state of the ionic grafts likely facilitates more charge transfer interactions with the excited states [44,45].

Here, the lower quantum yields of the PSPA grafted polymer indicates that more non-radiative pathways are available for the excited state’s decaying, indicating that either it is more tightly aggregated in solution compared to the PMETAC grafted polymer or that there is a higher degree of charge transfer of the excited state to the ionic side chains.

**Figure 4:**
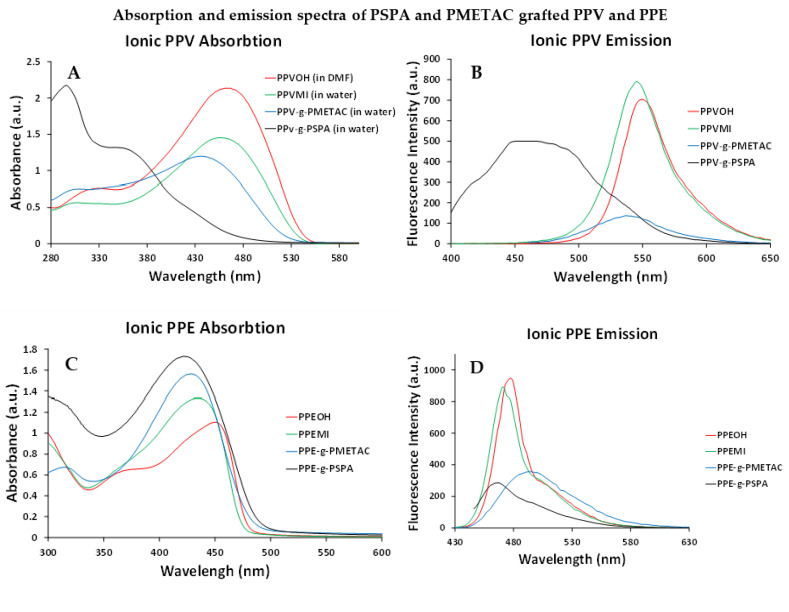
Absorption and emission spectra for ionic PPVs (**A**,**B**) and ionic PPEs (**C**,**D**) with ionic polymers in water and the backbone in DMF.

In most of the cases, the PPEs follow the same trend as the PPVs, with a blue shift in the absorption and reduction in quantum yield. The magnitude of the shifts for the PPEs is not to the same extents as that for PPV, but this is expected due to the PPE’s superior stability, and thus, fewer defects are added. The one exception to the blue shifting trend is the emission spectra for **PPE-*g*-PMETA** where there is a red shift in the emission spectra (Figure 4). To understand this, we must first look at what causes self-quenching and a red shift (bathochromic). Fluorescence self-quenching occurs when the molecules come within close proximity of each other (aggregate), and when there are more systems of similar energy that are within adequate proximity for inter-system crossing, internal conversion and relaxation, and thus, there are higher chances for non-radiative decay, which is best depicted with a Jablonski diagram (See Supporting Information Figure S1) [46,47,48]. This is generally seen with a red shift and broadening in the fluorescence, as more lower-energy (higher wavelength) radiative decay pathways are also made available [47,48,49]. In all the cases so far, where self-quenching is observed (**PPV-*g*-P*n*BA**, **PPV-*g*-PPMETAC** and **PPV-*g*-PSPA**), the expected red shift has been offset by the blueshift caused by the added defects. Evidence for this is provided by comparing the Stokes shift changes when comparing the **PPVOH** (or **PPEOH**) and **PPVMI** (or **PPEMI**) and that of the different copolymers and the copolymers graft lengths. In all cases, the transition from alcohol-functionalized polymer to macroinitiator is met with a small blue shift in the adsorption and the emission spectra, resulting in a similar Stokes shift between the macroinitiator and the alcohol-functionalized polymer. However, when the polymers are then grafted upon, the resulting blue shift in the adsorption does not coincide with as large a blue shift as in the emission spectra, and thus, the resulting Stokes shift is larger. Thus, this is an indication that there is a competing red shift for the copolymer’s emission spectra. The relatively small blue shift of 7 nm from ungrafted **PPEMI** to grafted **PPE-*g*-PMETAC** then explains the 17 nm red shift for the fluorescence emission spectra, where aggregation is causing emission bathochromatism, but the defects are causing absorption hypsochromatism.

In all graft copolymers with ionic side chains, it was observed that the quantum yield decreases with the increased graft lengths and that the maximum emission wavelength is further red shifted.

**Figure 5:**
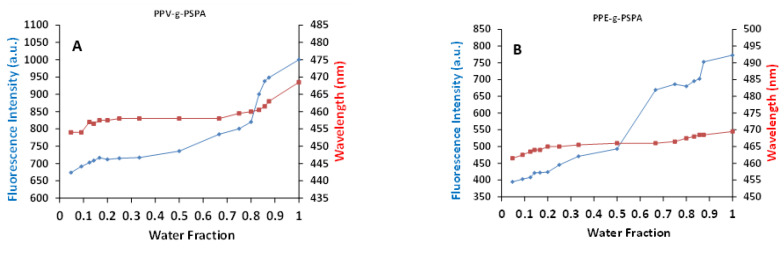
Fluorescence intensity and corresponding fluorescence maximum wavelength shifts for the anionic (PSPA) grafted PPVs (**A**) and PPEs (**B**) with shifting volume fraction of water in methanol.

The results above emphasize the strong interdependences between the molecular architecture of the graft copolymers, their intermolecular interactions and the interactions with the media, on their optical properties. To demonstrate this further, the fluorescence intensity (which are proportional to quantum yield at constant chromophore concentrations) and maximum emission wavelength were tracked while varying solvent polarity (with water/methanol mixes) (Figure 5). The fluorescence intensity for the PSPA grafted polymers shifted as much as 33% for **PPV-*g*-PSPA** and 51% for **PPE-*g*-PSPA**. The intensity increased sharply at specific water fractions (0.8 for the PPV and 0.5 for the PPE), indicating the collapse of aggregates. The PMETAC grafted polymers also showed a solvent polarity effect; however, the effects were far more erratic. This indicates that the aggregation induced by solvent polarity was very sensitive, forming different structures with slight changes to the solvent environment. For these PMETAC grafted copolymers, the intensity changed up to 28% for **PPV-*g*-PMETAC** and 64% for **PPE-*g*-PMETAC**.

From a molecular engineering approach, these results demonstrate the care needed in order to control the physical and optical properties of conjugated polymers. While the physical properties of the polymers can be manipulated through lateral grafting, how these polymers behave in solution strongly dictates their optical properties. Here, and previously, we have shown that neutral grafts of P*n*BA can facilitate a more processable conjugated polymer, where the physical properties are dominated by that of the lateral grafts. This is extremely useful considering the limited processability of conjugated polymers. However, for introducing ionic, and therefore hydrophilic, grafts, these will have an opposing hydropathy to the backbone polymer. As demonstrated, this creates a sensitive system where the solvent polarity can influence the polymers morphology in water and thus influence the optical properties. Such systems would show great promise in the fabrication of optical sensing devices.

## 4. Conclusions

The effects of grafting of uncharged, cationic and anionic polymer brushes from PPV and PPE backbones have been compared. The kinetic studies showed that the ATRP grafting polymerization was not fully controlled; however, it was sufficient to prepare copolymers of different graft lengths to be examined for the effect of the grafts on the copolymers optical properties. Insights from the ATRP kinetics reveal the effects the grafts have on the polymer’s solubility and how the copolymers are behaving in their respective solutions. In general, the optical properties of PPVs show a large blue shift in both fluorescence and absorption after grafting. This is attributed to the addition of parylene-type defects to the PPV backbone during grafting. On the other hand, PPE did not show such a hypsochromatic shift and is attributed to the increased stability of the PPE backbone compared to the PPV backbone. The order of the magnitude of the adsorption hypochromatic shift caused by grafting follows PSPA > P*n*BA > PMETAC. The solution-state quantum yields indicate that large amounts of self-quenching is occurring for both ionic grafts of PPV in water, with **PPV-*g*-PSPA** having slightly more quenching, indicated by its lower quantum yield. Interestingly, PPEs do not show the same behavior as PPVs. Here, we show that PPE is more robust against radical attacks as there is very little blue shift caused by the grafting of any of the investigated grafts; however, similarly to PPVs, large amounts of self-quenching was evident for the ionic grafted PPEs in water, with the three grafts showing a solution-state quantum yield of 90, 25 and 25% for the neutral grafts in DMF, the PMETAC cationic grafts in water and the PSPA anionic grafts in water, respectively. The self-quenching of ionic grafted polymers in water is attributed to the formation of aggregates, polymer structural conformation and more non-radiative charge transfer pathways, and it is confirmed through systematically changing the aquesous solutions polarity by the gradual addition of methanol. In doing so, the fluoresence intensity increases as the methanol content also increases. Such polymer systems have potential applications in optical hydropathy sensors, due to the drastic change in fluorescent intensity with small changes in solvent polarity.

## Figures and Tables

**Table 1:** R^2^ values for the ATRP kinetics of macroinitiators for neutral (P*n*BA), cationic (PMETAC) and anionic (PSPA) grafts. Values are determined through linear regression of the column titles against time. The R^2^ values closest to 1 are bolded for each row.

	R^2^ Values		
PPV	−Ln(M/M_0_) For 1st Order	1/[M] For 2nd Order	1/[M]^2^ For 3rd Order
P*n*BA	0.958	0.980	0.977
PMETAC	0.885	0.941	**0.976**
PSPA	**0.995**	0.983	0.952
PPE	**−Ln(M/M_0_)**	**1/[M]**	**1/[M]^2^**
P*n*BA	0.974	**0.997**	0.988
PMETAC	**0.987**	0.975	0.928
PSPA	**0.995**	0.989	0.968

**Table 2:** Absorption and emission max, stokes shift and quantum yields of PPVs and PPEs grafted with P*n*BA.

Polymer	λ_max abs_ (nm)	λ_max em_	Stokes Shift (nm)	Φ_solution_ (%)
**PPVOH**	461	550	89	53
**PPVMI**	451	545	94	59
**PPV-*g*-P*n*BA (4 h)**	378	489	111	30
**PPV-*g*-P*n*BA (8 h)**	378	497	119	71
**PPV-*g*-P*n*BA (24 h)**	378	502	124	86
**PPEOH**	450	478	28	32
**PPEMI**	435	473	38	87
**PPE-*g*-nBA (8 h)**	428	468	40	71
**PPE-*g*-nBA (24 h)**	428	473	45	90

**Table 3:** Absorption and emission maximum wavelengths, stokes shifts and quantum yields of PPVs and PPEs grafted with ionic PMETAC and PSPA side chains. LM_w_ and HM_w_ designates the graft copolymers formed from the monomer: macroinitiators (PPVMI/PPEMI) ratios of either 250:1 (LM_w_) or 500:1 (HM_w_).

Polymer	λ_max abs_ (nm)	λ_max em_ (nm)	Stokes Shift (nm)	Φ_solution_ (%)
**PPVOH**	458	550	92	53
**PPVMI**	456	545	89	59
**PPV-*g*-PMETAC (LM_w_)**	436	535	99	58
**PPV-*g*-PMETAC (HM_w_)**	436	537	101	32
**PPV-*g*-PSPA (LM_w_)**	349	470	121	43
**PPV-*g*-PSPA (HM_w_)**	349	470	121	17
**PPEOH**	435	477	42	32
**PPEMI**	428	471	43	87
**PPE-*g*-PMETAC (LM_w_)**	428	493	65	49
**PPE-*g*-PMETAC (HM_w_)**	428	497	69	25
**PPE-*g*-PSPA (LM_w_)**	422	470	48	52
**PPE-*g*-PSPA (HM_w_)**	422	474	52	25

## Data Availability

Not applicable.

## Supplementary Materials

The following supporting information can be downloaded at: https://www.mdpi.com/article/10.3390/polym14142767/s1, synthesis details for monomers and polymers and GPC chromatographs, Figure S1: Jablonksi diagram for fluorescent and phosphorescent compounds, Figures S2–S8: ^1^H NMR spectra of grafted polymers and ATRP monomers, Table S1: Molecular weight characteristics of synthesised polymers as determined by GPC and NMR kinetics and Scheme S1: Phase transfer complexation of ATRP monomer and 18-Crown-6.
