# Supplementary material for: Effects of Neutral, Anionic and Cationic Polymer Brushes Grafted from Poly(para-phenylene vinylene) and Poly(para-phenylene ethynylene) on the Polymer’s Photoluminescent Properties
Source: Polymers (Basel). 2022 Jul 6;14(14):2767. doi: 10.3390/polym14142767 (PMC9322352; doi:10.3390/polym14142767)

Supporting Information:

Effects of Neutral, Anionic and Cationic Polymer Brushes Grafted from Poly(para-phenylene vinylene) and Poly(para-phenylene ethynylene) on the Polymer's Photoluminescent Properties

Thomas Kerr-Phillips [1,2,†], Mona Damavandi [1,2,†], Lisa I. Pilkington[1,2], Kathryn A. Whitehead,[3] Jadranka Travas-Sejdic [1,2] and David Barker [1,2, *]

[1] School of Chemical Sciences, University of Auckland, Private Bag, Auckland 92019, New Zealand
[2] The MacDiarmid Institute for Advanced Materials and Nanotechnology, Victoria University of Wellington, Wellington 6012, New Zealand
[3] Microbiology at Interfaces, Manchester Metropolitan University, Chester Street, Manchester M1 5GD, UK
* Correspondence d.barker@auckland.ac.nz; Tel.: +64 9 373 7599
† These authors contributed equally.

Polymer Synthesis via ATRP:

Synthesis of PPV-g-nBA via ARGET ATRP

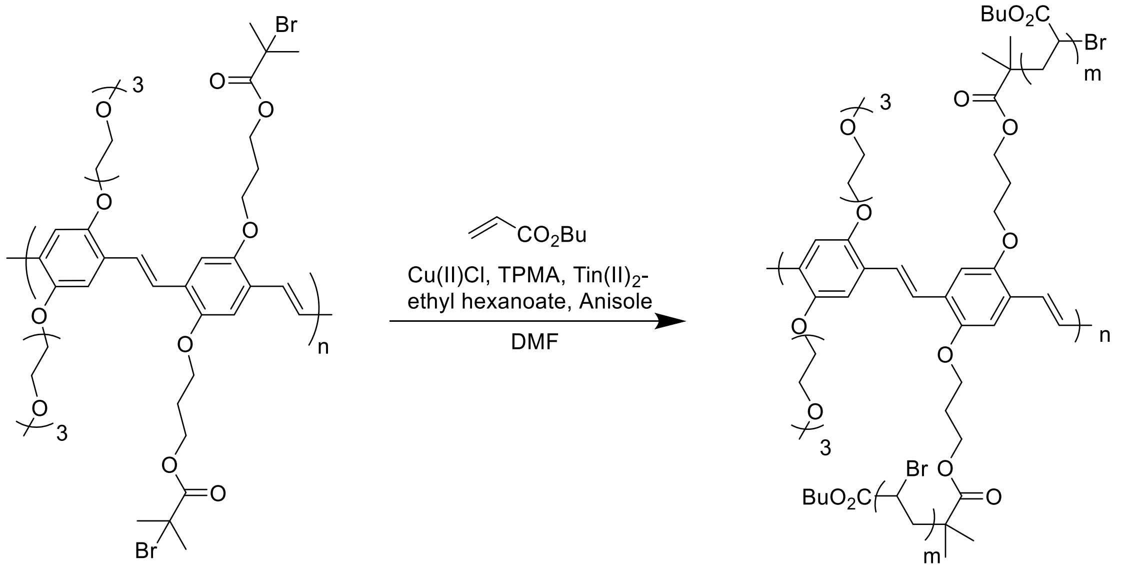

A two-neck flask was loaded with a mixture of PPV-MI (270 mg, 0.02 mmol), anisole (250 μL), n-BA (1.76 mL, 12.27 mmol) and DMF (10 mL) under a nitrogen atmosphere. The mixture was then degassed using the freeze-thaw method with nitrogen, twice. A solution of Cu(II) complex tri(2-pyridylmethyl)amine (TPMA) (6.5 mg, 0.022 mmol) in DMF (1 mL), was prepared at 45 °C for 3 h and then added to the mixture. The reaction was then stirred at 65 °C. Tin(II) ethylhexanoate (0.3 mL, 0.93 mmol) was then added and the mixture stirred for 24 h at 65 °C, under an atmosphere of nitrogen. The solution was allowed to cool to room temperature and then dissolved in CH_2Cl_2, reduced via vacuum, precipitated by the addition of methanol (100 mL) and centrifuged (40 rpm, 15 min) to collect the precipitate. The precipitate was dialysed with dialysis bag (Sigma dialysis tubing 10 mm) using methanol. The precipitate in the dialysis bag was then collected and dried under vacuum to afford the desired PPV-g-nBA 4.1 as a bright yellow gum (0.8 g, 60 %).

δH (400 MHz; $CDCl_3$; Me_4Si): 0.92-0.95 (3H, m, CH_3), 1.34-1.40 (2H, m, CH_2), 1.58-1.63 (2H, m, CH_2), 1.75 (1H, m, CH), 1.91-2.30 (2H, m, CH_2), 4.08-4.11 (2H, m, CH_2). GPC: Mw: 8.9×104 gmol-1, Mn: 6.3×104 gmol-1, Mw/Mn: 1.41. UV (4 mgmL-1, DMF) λmax abs = 378 nm. PL (350 µgmL-1, DMF) λmax em = 501 nm.

Synthesis of PPE-g-nBA via ARGET ATRP

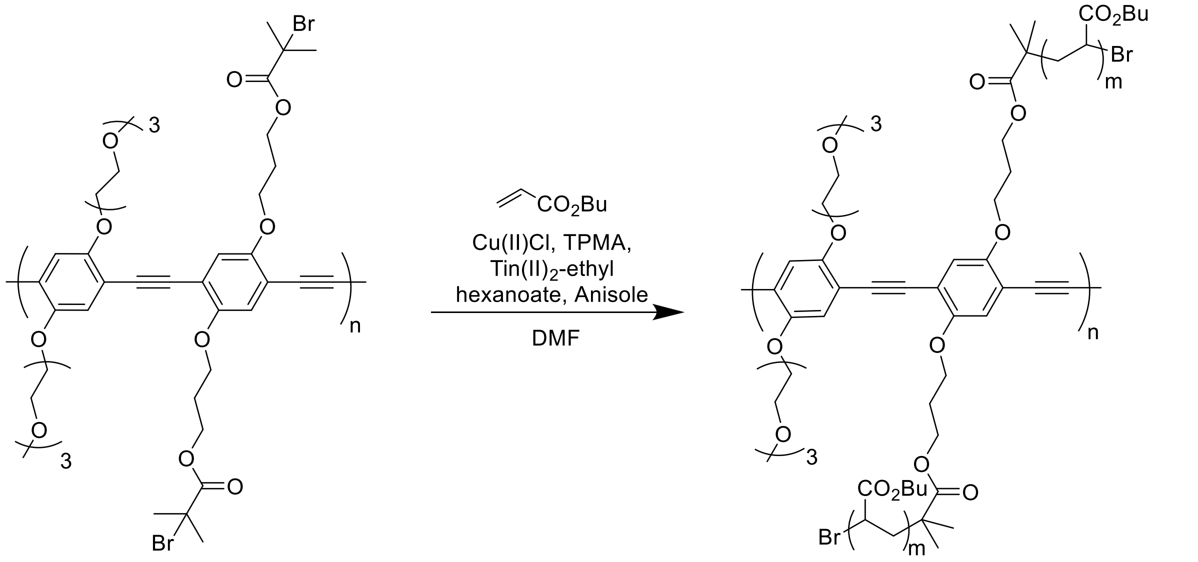

A two-neck flask was loaded with a mixture of PPE-MI 3.2.2 (220 mg, 0.02 mmol), anisole (250 µL), nBA (1.76 mL, 12.27 mmol) in DMF (10 mL) under a nitrogen atmosphere. The mixture was then degassed using the freeze-thaw method with nitrogen, twice. A solution of Cu(II) complex tri(2-pyridylmethyl)amine (TPMA) (6.5 mg, 0.0224 mmol) in DMF (1 mL), was prepared at 45 °C for 3 h and then added to the mixture. The reaction was then stirred at 65 °C. Tin(II) ethylhexanoate (0.3 mL, 0.93 mmol) was then added and the mixture stirred for 24 h at 65 °C, under an atmosphere of nitrogen. The solution was allowed to cool to room temperature and then dissolved in CH_2Cl_2, reduced via vacuum, precipitated by the addition of methanol (100 mL) and centrifuged (40 rpm, 15 min) to collect the precipitate. The precipitate was dialysed with dialysis bag (Sigma dialysis tubing 10 mm) over methanol. The precipitate in the dialysis bag was then collected and dried under vacuum to afford the desired PPE-g-PnBA 4.2 as a bright yellow gum (0.9 g, 62 %).

δH (400 MHz; $CDCl_3$; Me_4Si): 0.91-0.97 (3H, m, CH_3), 1.36-1.41 (2H, m, CH_2), 1.59-1.63 (2H, m, CH_2), 1.78 (1H, m, CH), 2.02-2.31 (2H, m, CH_2), 4.10-4.14 (2H, m, CH_2). GPC: Mw: 7.4×104 gmol-1, Mn: 5.5×104 gmol-1, Mw/Mn: 1.35. UV (4 mgmL-1, DMF) λmax abs = 422 nm. PL (350 µgmL-1, DMF) λmax em = 473 nm.

Synthesis of Microinitiator-g-PMETAC via ARGET ATRP

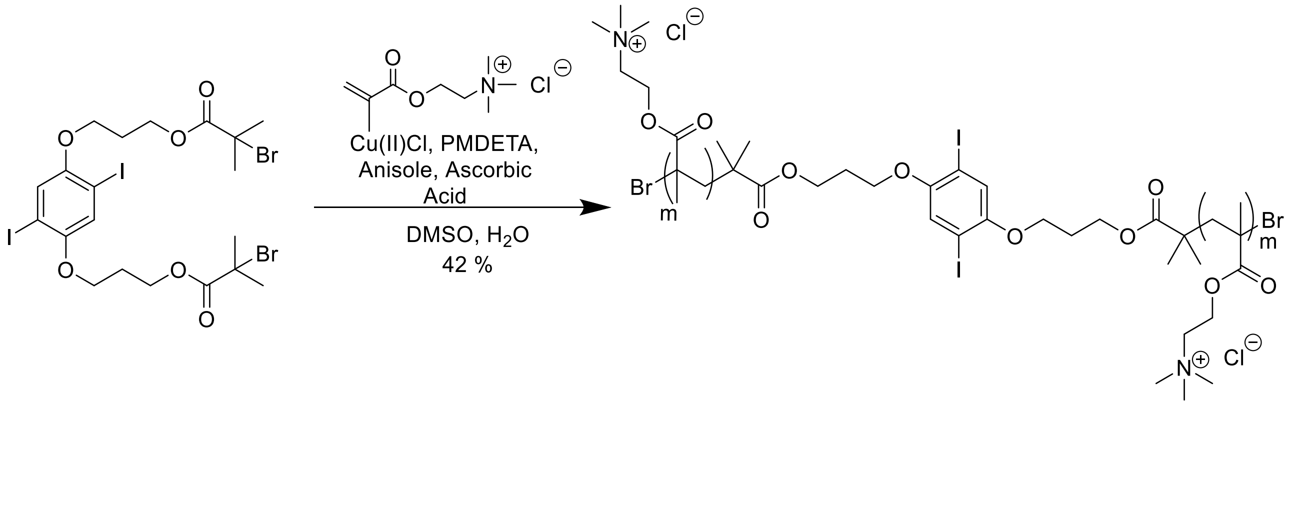

A solution of monomer microinitiator (15 mg, 0.02 mmol) in DMSO (5 mL) was added to a stirring solution of the 2-(methacryloyloxy)ethyl]trimethylammonium chloride solution(1.88 mL, 10 mmol) in DMSO (5 mL) and water (600 μL) to achieve a colourless solution. Separately, the ligand-catalyst complex was prepared by adding PMDETA (7.5 mg, 0.041 mmol) into a mixture of Cu(II)Cl (2 mg, 0.0148 mmol) and anisole (1 mL) at 67 °C for 3 h. This complex was added to the reaction mixture at 60 °C. Then, a solution of ascorbic acid (980 mg, 5.56 mmol) in anisole (1 mL) and water (300 μL) was added slowly to the reaction and left for 24 h under an atmosphere of nitrogen at 60 °C. The reaction was quenched by cooling the mixture with liquid nitrogen and exposing the mixture to air. The white precipitate was collected and then dissolved in water. The product was re-precipitated using acetone (100 mL) and collected using centrifuge to achieve polymer as a white solid (78 mg, 42 %).

δH (400 MHz; D_2O): 1.01-1.12 (2H, m, CH_2), 1.97-2.03 (1H, m, CH), 3.27 (9H, br s, CH_3), 3.78-3.83 (2H, m, CH_2), 4.48-4.52 (2H, m, CH_2). GPC: Mw: 6.41×103 gmol-1, Mn: 4.98×103 gmol-1, Mw/Mn: 1.288.

Synthesis of high and molecular weight PPV-g-PMETAC via ARGET ATRP

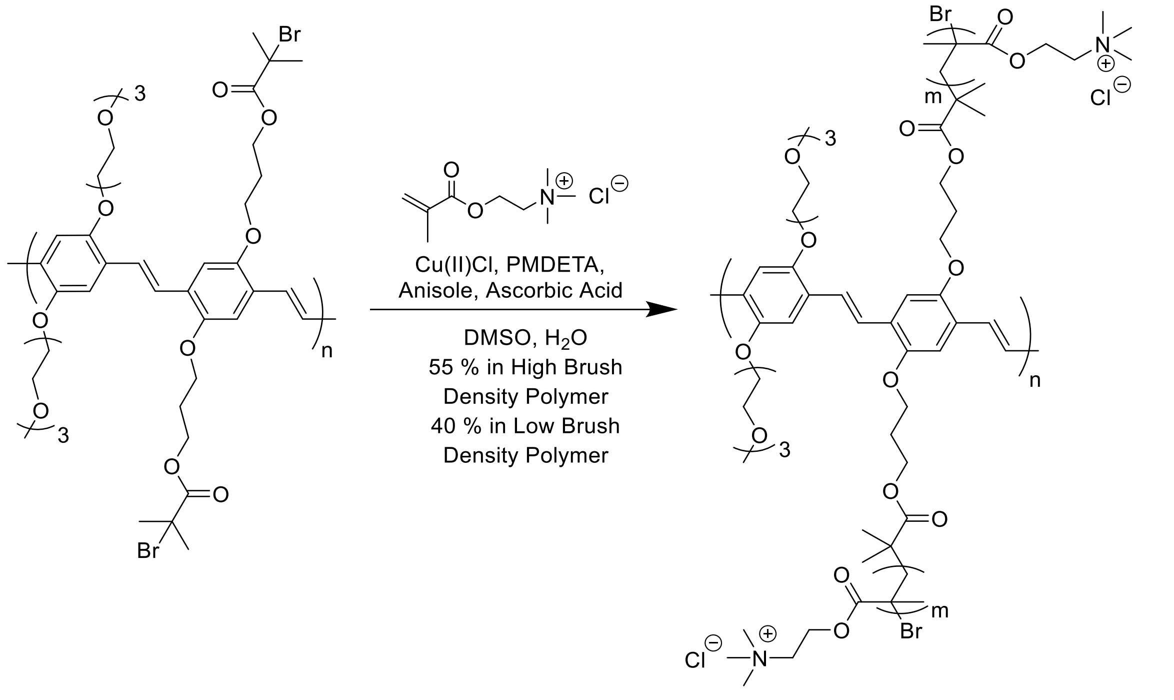

A solution of PPVMI (270 mg, 0.01 mmol) in DMSO (5 mL) was added to a stirring solution of the 2-(methacryloyloxy)ethyl]trimethylammonium chloride solution (1.88 mL, 10 mmol) in DMSO (5 mL) and water (600 μL) to achieve a colourless solution. Separately, the ligand-catalyst complex was prepared by adding PMDETA (7.5 mg, 0.041 mmol) into a mixture of Cu(II)Cl (2 mg, 0.0148 mmol) mixture in anisole (1 mL) at 67 °C for 3 h. This complex was added to the reaction mixture at 60 °C. Then a solution of ascorbic acid (980 mg, 5.56 mmol) in anisole (1 mL) and water (300 μL) was added slowly to the reaction and left for 24 h under an atmosphere of nitrogen at 60 °C. The reaction was quenched by cooling the mixture with liquid nitrogen and exposing the mixture to air. The orange precipitate was collected and then dissolved in water. The product was re-precipitated using acetone (100 mL) and collected using centrifuge to give polymer a bright orange solid (850 mg, 55 %).

δH (400 MHz; D_2O): 1.01-1.10 (2H, m, CH_2), 1.95-2.01 (1H, m, CH), 3.25 (9H, br s, CH_3), 3.75-3.82 (2H, m, CH_2), 4.45-4.50 (2H, m, CH_2). GPC: Mw: 77.49×103 gmol-1, Mn: 46.19×103 gmol-1, Mw/Mn: 1.67. UV (2 mgmL-1, water) $\lambda_{max\ abs}$ = 435 nm. PL (125 μgmL-1, water) $\lambda_{max\ em}$ = 535 nm.

The same procedure was used to achieve the low molecular weight PPV-g-PMETAC, except using 2-(methacryloyloxy)ethyl]trimethylammonium chloride solution (950 μL, 5 mmol) in DMSO (5 mL) and water (300 μL) and ascorbic acid (600 mg, 3.40 mmol) in anisole (1 mL) and water (150 μL) to give polymer as a bright orange solid (180 mg, 40 %).

δH (400 MHz; D_2O): 1.01-1.10 (2H, m, CH_2), 1.92-2.03 (1H, m, CH), 3.28 (9H, br s, CH_3), 3.77-3.85 (2H, m, CH_2), 4.43-4.48 (2H, m, CH_2). GPC: Mw: 22.86×103 gmol-1, Mn: 16.72×103 gmol-1, Mw/Mn: 1.36. UV (2 mgmL-1, water) $\lambda_{max\ abs}$ = 435 nm. PL (125 μgmL-1, water) $\lambda_{max\ em}$ = 537 nm.

Synthesis of high and low molecular weight PPE-g-PMETAC via ARGET ATRP

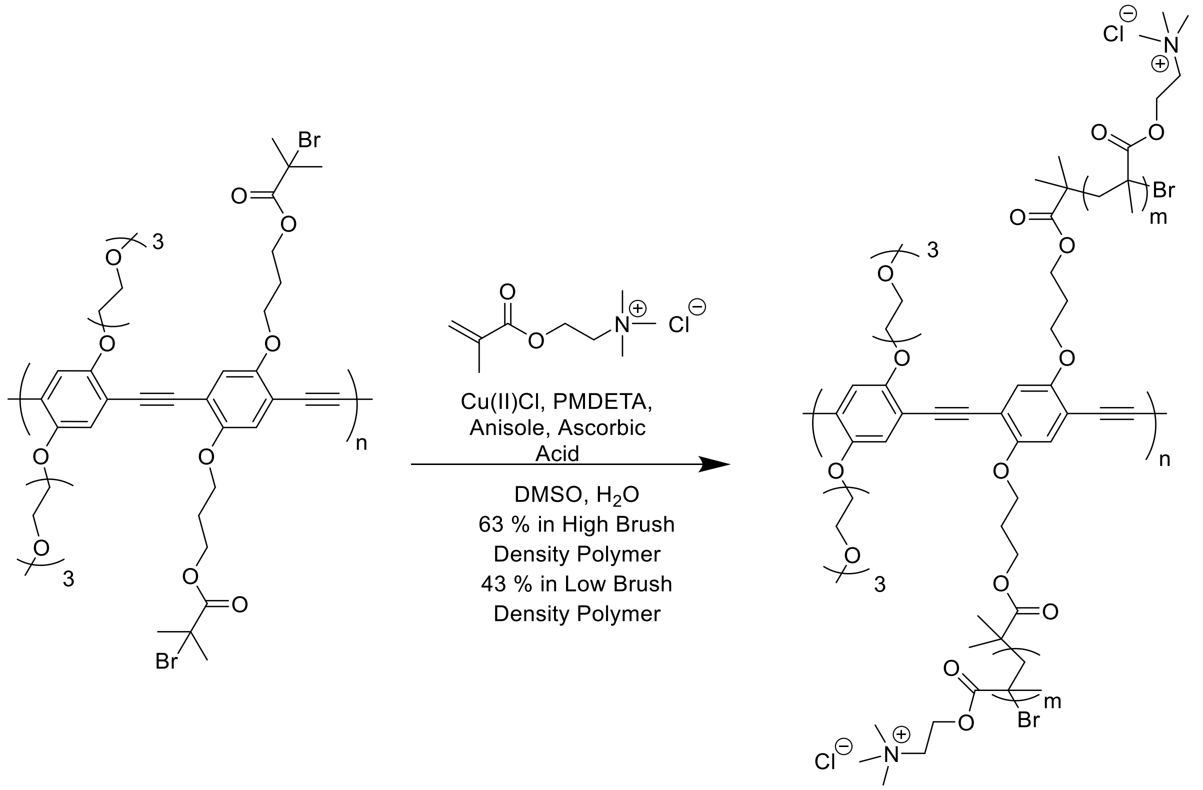

A solution of PPEMI (220 mg, 0.02 mmol) in DMSO (5 mL) was added to a stirring solution of the 2-(methacryloyloxy)ethyl]trimethylammonium chloride solution (1.88 mL, 10 mmol) in DMSO (5 mL) and water (600 μL) to achieve a colourless solution. Separately, the ligand-catalyst complex was prepared by adding PMDETA (7.5 mg, 0.041 mmol) into a mixture of Cu(II)Cl (2 mg, 0.0148 mmol) in anisole (1 mL) at 67 °C for 3 h. This complex was added to the reaction mixture at 60 °C. Then a solution of ascorbic acid (980 mg, 5.56 mmol) in anisole (1 mL) and water (300 μL) was added slowly to the reaction and left for 24 h under an atmosphere of nitrogen at 60 °C. The reaction was quenched by cooling the mixture with liquid nitrogen and exposing the mixture to air. The orange precipitate was collected and then dissolved in water. The product was re-precipitated using acetone (100 mL) and collected using centrifuge to give polymer as a bright orange solid (870 mg, 63 %).

δH (400 MHz; D_2O): 1.02-1.11 (2H, m, CH_2), 1.97-2.03 (1H, m, CH), 3.26 (9H, br s, CH_3), 3.77-3.82 (2H, m, CH_2), 4.47-4.51 (2H, m, CH_2). GPC: Mw: 77.48×103 gmol-1, Mn: 46.02×103 gmol-1, Mw/Mn: 1.68. UV (5 mgmL-1, water) $\lambda_{max\ abs}$ = 428 nm. PL (5 mgmL-1, water) $\lambda_{max\ em}$ = 493 nm.

The same procedure was used to achieve the low molecular weight PPE-g-PMETAC, except using 2-(methacryloyloxy)ethyl]trimethylammonium chloride solution (950 µL, 5 mmol) in DMSO (5 mL) and water (300 µL) and a solution of ascorbic acid (600 mg, 3.40 mmol) in anisole (1 mL) and water (150 µL) to give polymer as a bright orange solid (190 mg, 43 %).

δH (400 MHz; D_2O): 1.04-1.13 (2H, m, CH_2), 1.95-2.01 (1H, m, CH), 3.24 (9H, Br s, CH_3), 3.78-3.83 (2H, m, CH_2), 4.45-4.47 (2H, m, CH_2). GPC: Mw: 22.43×103 gmol-1, Mn: 16.38×103 gmol-1, Mw/Mn: 1.37. UV (2 mgmL-1, water) $\lambda_{max\ abs}$ = 428 nm. PL (125 µgmL-1, water) $\lambda_{max\ em}$ = 497 nm.

Synthesis of High Molecular Weight Microinitiator-g-PSPA

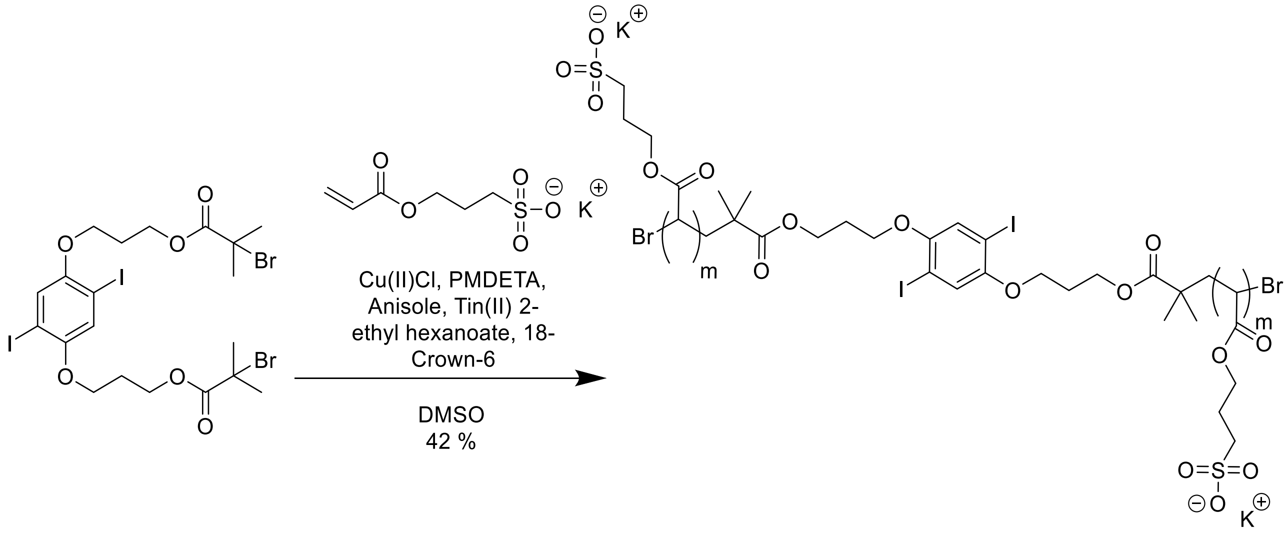

A solution of Microinitiator (15 mg, 0.02 mmol) in DMSO (5 mL) was added to a solution of 3-(acryloyloxy)propane-1-sulfonate (2.077 g, 10 mmol) and 18-Crown-6 (2.65 g, 10.1 mmol) in DMSO (10 mL). Separately the ligand-catalyst complex was prepared by adding PMDETA (7.5 mg, 0.041 mmol) to a solution of Cu(II)Cl (2 mg, 0.0148 mmol) in anisole (1 mL) and stirring the mixture at 67 °C for 3 hours. This solution was added to the reaction mixture at 60 °C. A solution of tin (II) 2-ethylhexanoate (1.8 mL, 5.56 mmol) in anisole (1 mL) was added slowly to the reaction and the mixture left for 24 h under an atmosphere of nitrogen, at 60 °C. The reaction was quenched by cooling the mixture with liquid nitrogen and exposing the mixture to air. A white precipitate was collected and then dissolved in water. The product was re-precipitated using acetone (100 mL), collected with centrifuge and purified using dialysis with 1,4-dioxane, water and KCl (2 M) to achieve a white solid (78 mg, 42 %).

δH (400 MHz; D_2O): 1.11-1.90 (2H, m, CH_2), 1.98-2.12 (2H, m, CH_2), 2.27-2.50 (1H, m, CH), 2.82-3.02 (2H, m, CH_2), 4.02-4.30 (2H, m, CH_2). GPC: Mw: 21.97×103 gmol-1, Mn: 16.20×103 gmol-1, Mw/Mn: 1.35.

Synthesis of High Molecular Weight and Low Molecular Weight PPV-g-PSPA

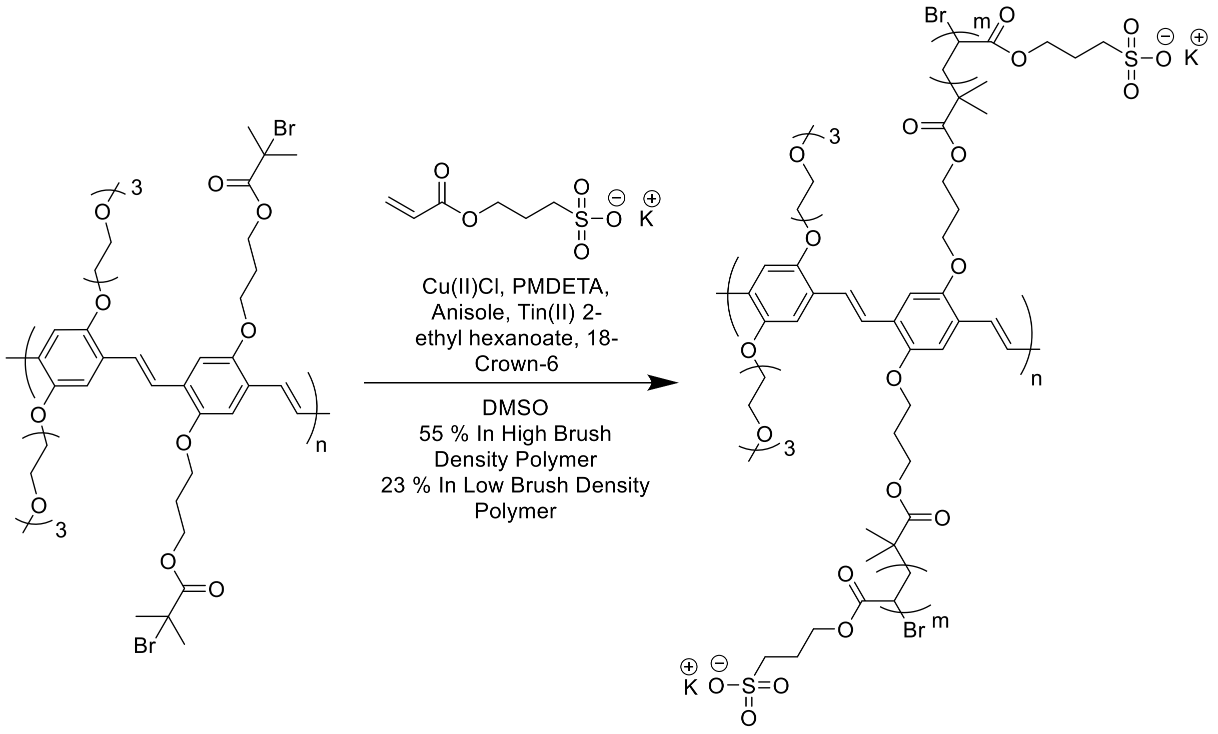

A solution of PPVMI (270 mg, 0.02 mmol) in DMSO (5 mL) was added to a solution of 3-(acryloyloxy)propane-1-sulfonate (2.08 g, 10 mmol) and 18-Crown-6 (2.65 g, 10.1 mmol) in DMSO (10 mL). Separately, the ligand-catalyst complex was prepared by adding the PMDETA (7.5 mg, 0.041 mmol) to a solution of Cu(II)Cl (2 mg, 0.0148 mmol) in anisole (1 mL) and stirring the mixture at 67 °C for 3 h. This complex was added to the reaction mixture at 60 °C. A solution of tin(II)2-ethylhexanoate (1.8 mL, 5.56 mmol) in anisole (1 mL) was added slowly to the reaction and the mixture left for 24 h under an atmosphere of nitrogen at 60 °C. The reaction was quenched by cooling the mixture with liquid nitrogen and exposing the mixture to air. An orange precipitate was collected and then dissolved in water. The product re-precipitated using acetone (100 mL), collected with centrifuge and purified using dialysis with 1,4-dioxane, water and KCl (2 M) to achieve polymer as a bright orange solid (430 mg, 55 %).

δH (400 MHz; D_2O): 1.14-1.92 (2H, m, CH_2), 2.02-2.14 (2H, m, CH_2), 2.29-2.51 (1H, m, CH), 2.85-3.04 (2H, m, CH_2), 4.05-4.33 (2H, m, CH_2). GPC: Mw: 78.08×103 gmol-1, Mn: 46.19×103 gmol-1, Mw/Mn: 1.72. UV (3.5 mgmL-1, water) $\lambda_{max\ abs}$ = 357 nm. PL (175 µgmL-1, water) $\lambda_{max\ em}$ = 470 nm.

The same procedure was used to achieve the low molecular weight PPV-g-PSPA, except using 3-(acryloyloxy)propane-1-sulfonate (1.04 g, 5 mmol) and 18-Crown-6 (1.32 g, 5 mmol) in DMSO (5 mL) and a solution of tin(II)2-ethylhexanoate (1100 µL, 3.40 mmol) in anisole (1 mL) to achieve polymer as a bright orange solid (53 mg, 23 %).

δH (400 MHz; D_2O): 1.12-1.12 (2H, m, CH_2), 2.03-2.15 (2H, m, CH_2), 2.31-2.61 (1H, m, CH), 2.85-3.04 (2H, m, CH_2), 4.07-4.38 (2H, m, CH_2). GPC: Mw: 22.87×103 gmol-1, Mn: 16.72×103 gmol-1, Mw/Mn: 1.37. UV (3.5 mgmL-1, water) $\lambda_{max\ abs}$ = 357 nm. PL (175 µgmL-1, water) $\lambda_{max\ em}$ = 470 nm.

Synthesis of High Molecular Weight 6.5 and Low Molecular Weight PPE-g-PSPA

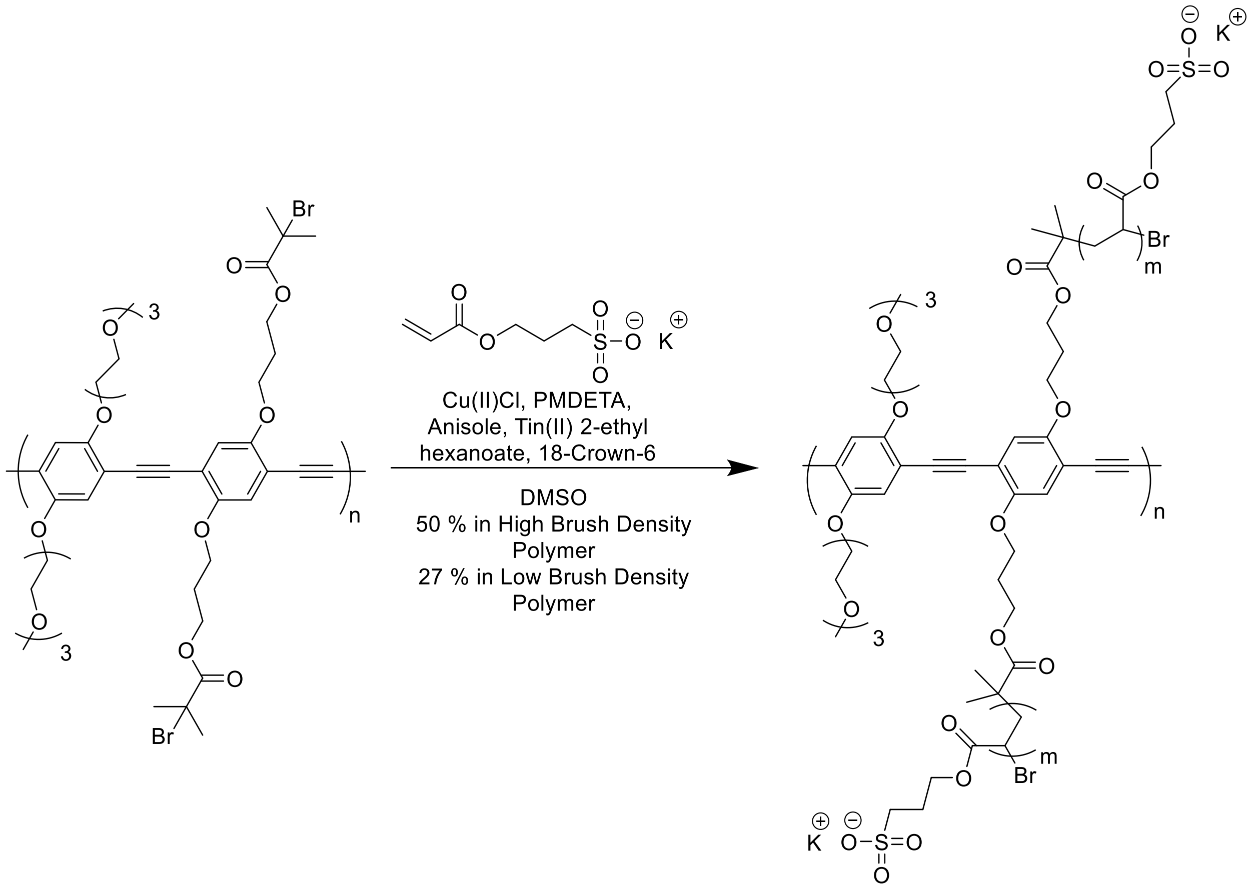

A solution of PPEMI (220 mg, 0.02 mmol) in DMSO (5 ml) was added to a stirring solution of the 3-(acryloyloxy)propane-1-sulfonate (2.077 g, 10 mmol) and 18-Crown-6 (2.65 g, 10.1 mmol) in DMSO (10 mL). Separately, the ligand-catalyst complex was prepared by adding PMDETA (7.5 mg, 0.041 mmol) to a solution of Cu(II)Cl (2 mg, 0.0148 mmol) in anisole (1 mL) and stirring the mixture at 67 °C for 3 h. This complex was added to the reaction mixture at 60 °C. A solution of tin(II)2-ethylhexanoate (1.8 mL, 5.56 mmol) in anisole (1 mL) was added slowly to the reaction and the mixture left for 24 h under an atmosphere of nitrogen, at 60 °C. The reaction was quenched by cooling the mixture with liquid nitrogen and exposing the mixture to air. The yellow precipitate was collected and then dissolved in water. The product was re-precipitated using acetone (100 mL), collected with centrifuge and purified using dialysis with 1,4-dioxane, water and KC (2 M) to achieve polymer as a bright yellow solid (387 mg, 50 %).

δH (400 MHz; D_2O): 1.12-1.90 (2H, m, CH_2), 1.98-2.12 (2H, m, CH_2), 2.27-2.48 (1H, m, CH), 2.82-3.02 (2H, m, CH_2), 4.03-4.31 (2H, m, CH_2). GPC: Mw: 77.48×103 gmol-1, Mn: 45.89×103 gmol-1, Mw/Mn: 1.68. UV (3.5 mgmL-1, water) $\lambda_{max\ abs}$ = 422 nm. PL (175 µgmL-1, water) $\lambda_{max\ em}$ = 474 nm.

The same procedure was used to achieve the low molecular weight PPE-g-PSPA 6.4, except using 3-(acryloyloxy)propane-1-sulfonate (1.04 g, 5 mmol) and 18-Crown-6 (1.32 g, 5 mmol) in DMSO (5 mL) and a solution of tin(II)2-ethylhexanoate (1100 µL, 3.40 mmol) in anisole (1 mL) to achieve polymer as a bright yellow solid (61 mg, 27 %).

δH (400 MHz; D_2O): 1.17-1.95 (2H, m, CH_2), 2.05-2.15 (2H, m, CH_2), 2.32-2.54 (1H, m, CH), 2.88-3.07 (2H, m, CH_2), 4.07-4.35 (2H, m, CH_2). GPC: Mw: 22.43×103 gmol-1, Mn: 16.38×103 gmol-1, Mw/Mn: 1.41. UV (3.5 mgmL-1, water) $\lambda_{max\ abs}$ = 422 nm. PL (175 µgmL-1, water) $\lambda_{max\ em}$ = 470 nm.

Molecular Weight Determination

The molecular weights of the prepared polymers was determined using gel permeation chromatography (GPC) and NMR. NMR derived molecular weights were determined by calculating the number of units consumed during the ATRP reactions and thus the molecular weights of the grafts, the GPC molecular weights of the backbone were then used with this to determine the overall molecular weights. For PPVOH, PPVMI, PPEOH and PPEMI the polymer's number-average molecular weight (M_n), average molecular weight (M_w) and the typical dispersity were measured using DMF as an eluent. Polystyrene standards were used for calibration and mass determination. Polystyrene were also used to assess the molecular weight of the PnBA grafted PPV and PPE, with THF as an eluent. For the ionic grafted polymers GPC was conducted using dextran standards in an aqueous solution of 0.02% NaN3.

Table S1 Molecular weight characteristics of synthesised polymers as determined by GPC and NMR kinetics.

Polymer	Mn	Mw(GPC)	Mw(NMR)	Mw / Mn
PPVOH	23100	37400		1.62
PPVMI	15400	22700		1.47
PPEOH	21700	28700		1.32
PPEMI	10700	18600		1.74
Direct Synthesised PPEMI	12000	22300		1.86
PPV-g-PnBA (4h)	21700	28700	32000	1.30
PPV-g-PnBA (8h)	19500	39500	40400	2.03
PPV-g-PnBA (24h)	63600	89900	64100	1.41
PPE-g-PnBA (4h)	22600	29100	30800	1.28
PPE-g-PnBA (8h)	41400	72000	42300	1.74
PPE-g-PnBA (24h)	55100	74300	64100	1.35
PPV-g-PMETAC (LMW)	16700	22900	21800	1.37
PPV-g-PMETAC (HMW)	46200	77500	75300	1.67
PPE-g-PMETAC (LMW)	16200	22000	22000	1.35
PPE-g-PMETAC (HMW)	45900	78100	78200	1.7
PPV-g-PSPA (LMw)	16700	22900	19900	1.37
PPV-g-PSPAA (HMw)	46200	78100	77600	1.72
PPE-g-PSPA (LMw)	16400	22400	22100	1.41
PPE-g-PSPAA (HMw)	45900	77500	75100	1.68

Phase transfer complexation (PTC) using 18-crown-6:

The PTC was used to improve SPA solubility by complexing the potassium ion (Scheme S1), and to improve the solubility of the grafted polymers in the DMSO solvent.

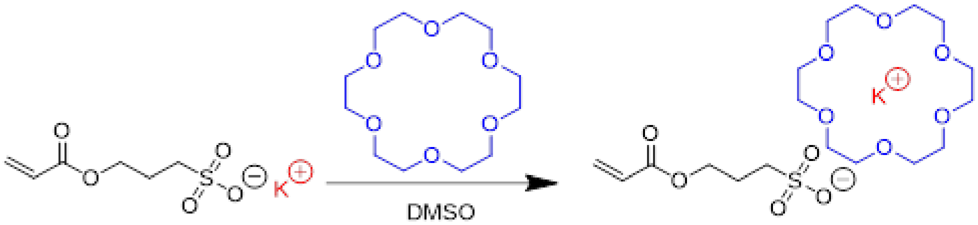

Scheme 1. Phase transfer complexation of ATRP monomer and 18-Crown-6.

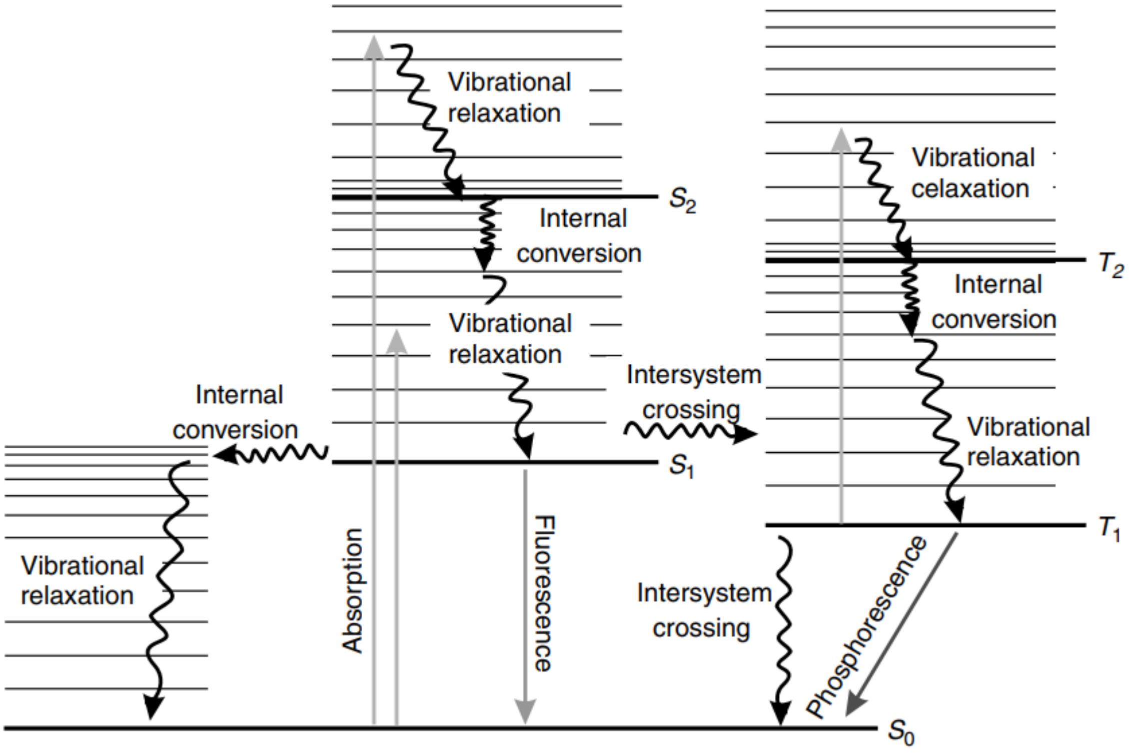

Figure S1. Jablonksi diagram for fluorescent and phosphorescent compounds.

NMR and GPC chromatograms

For the neutral P*n*BA grafted polymers, please see the supporting information of our previous work. [1]

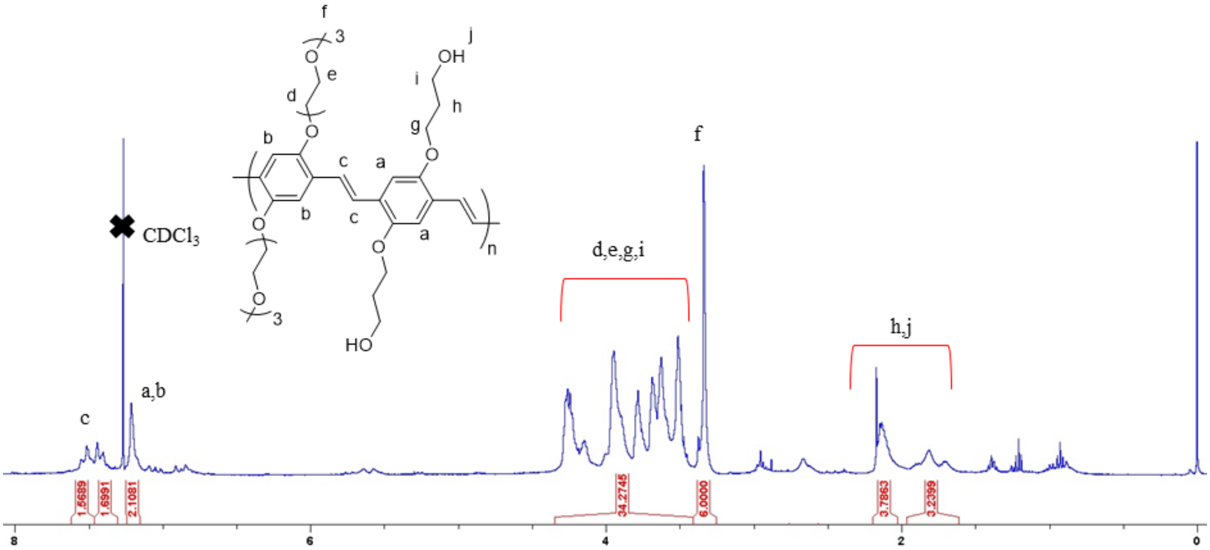

Figure S2.^{1}H NMR spectra of PPVOH in $CDCl_3$

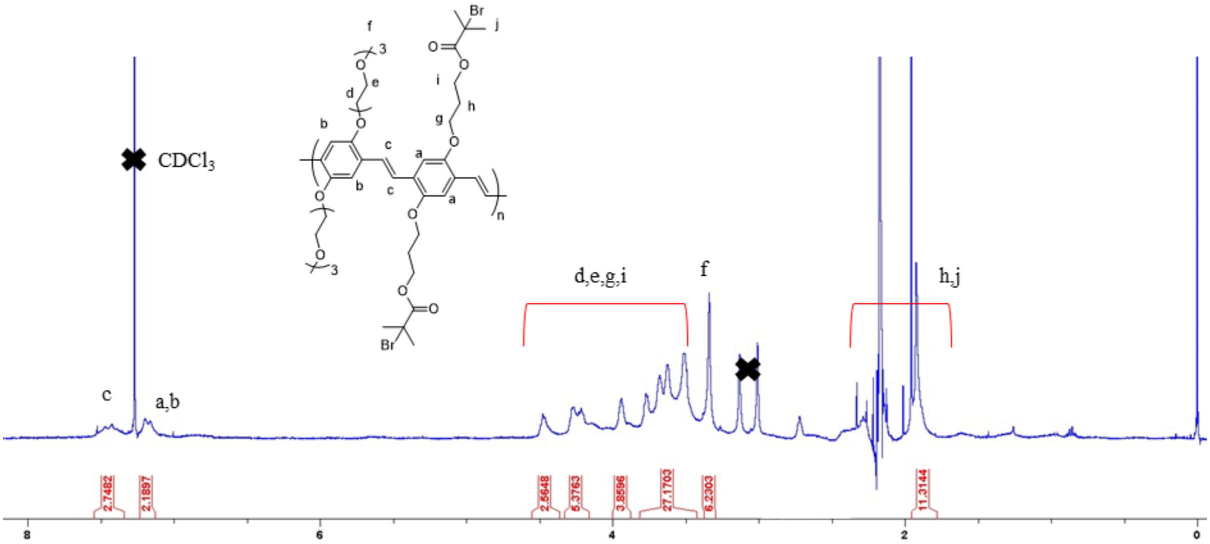

Figure S3 ^{1}H NMR spectra of PPVMI in $CDCl_3$

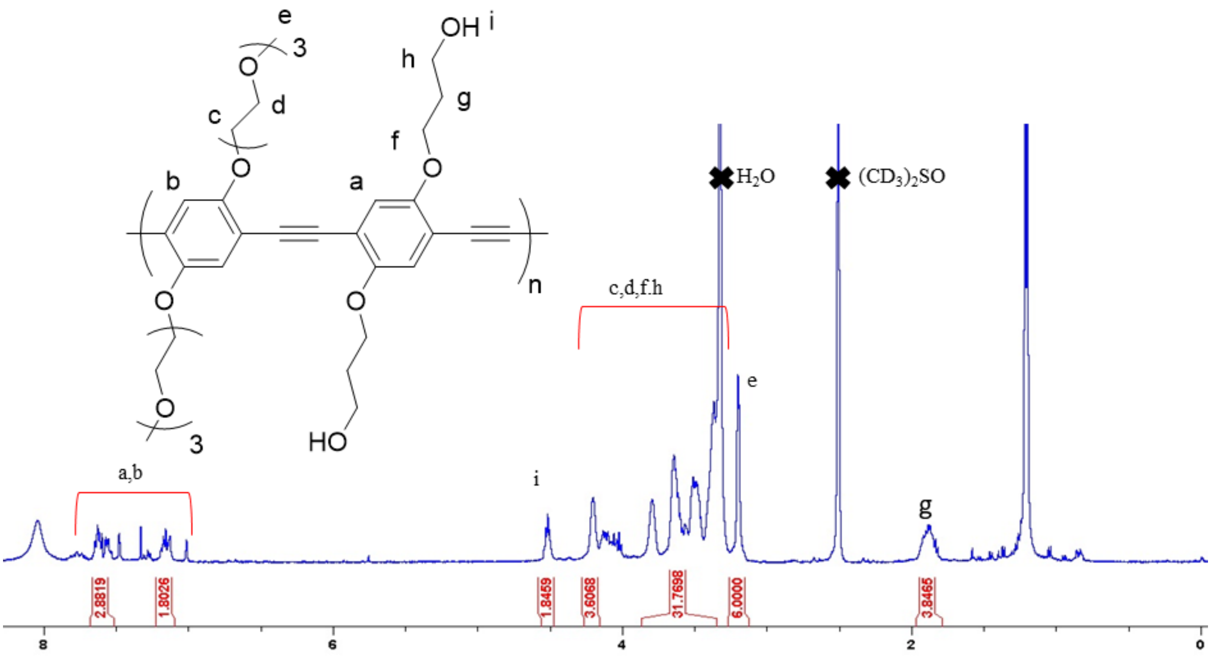

Figure S4. ^{1}H NMR spectra of PPEOH in DMSO

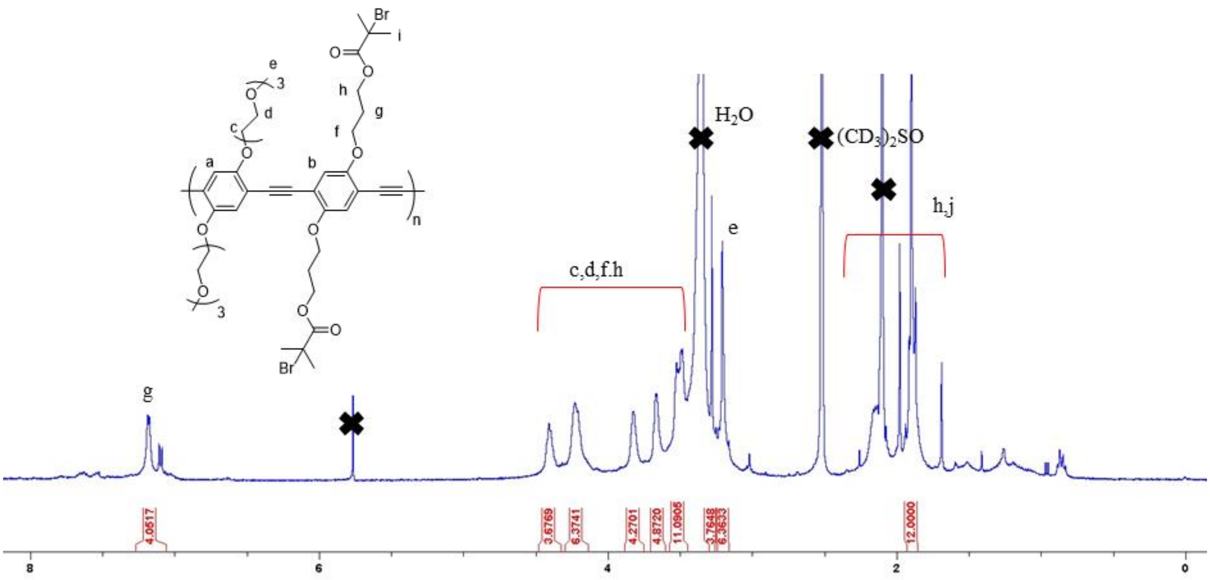

Figure S5. ^{1}H NMR spectra of PPEMI in DMSO.

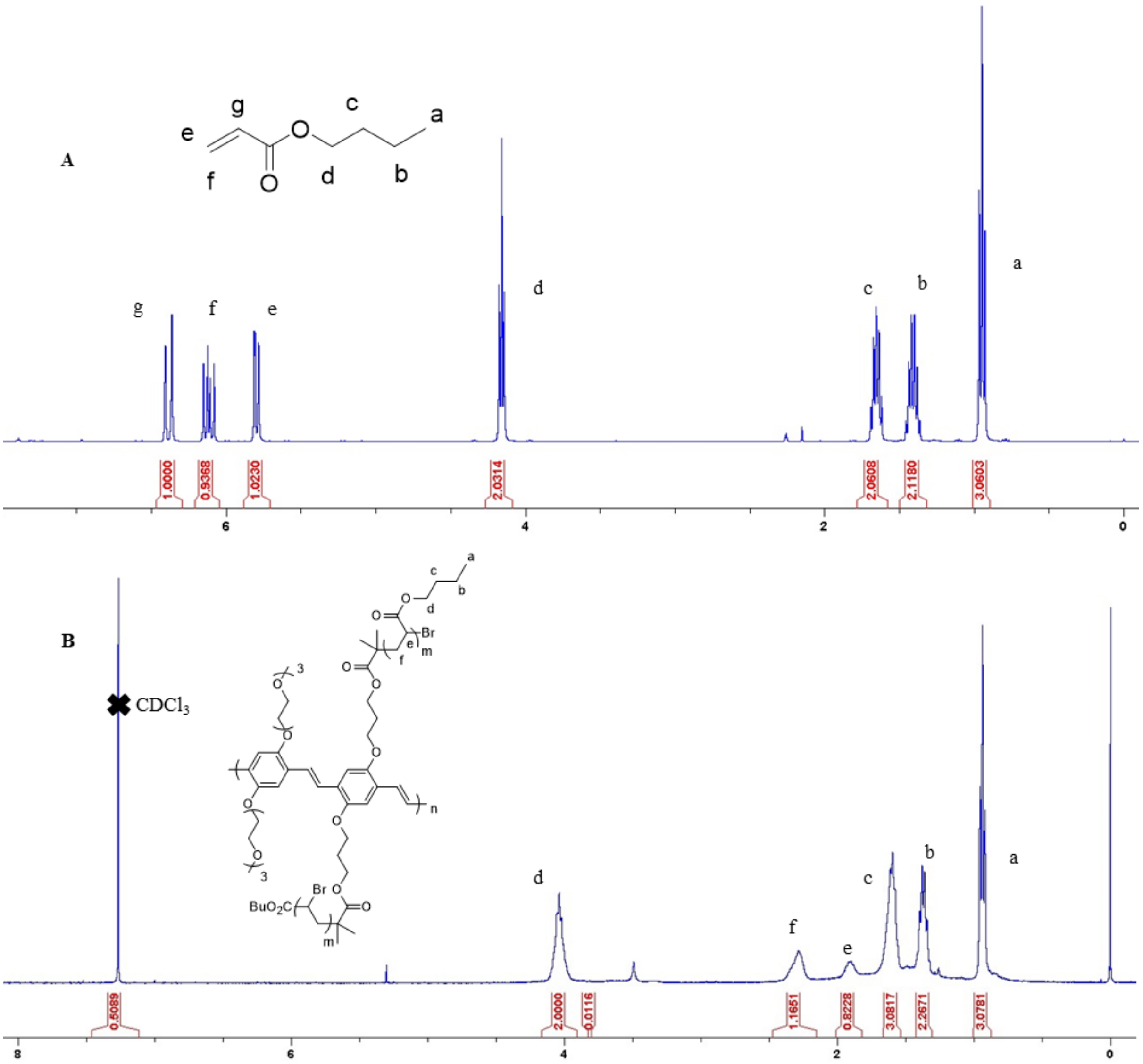

Figure S6. ^{1}H NMR spectra of *n*BA (A) and PPV-g-P*n*BA (B) in $CDCl_3$. The ^{1}H NMR spectra of PPE-g-P*n*BA is effectively identical to PPV-g-P*n*BA due to the high ratio of grafts to backbone.

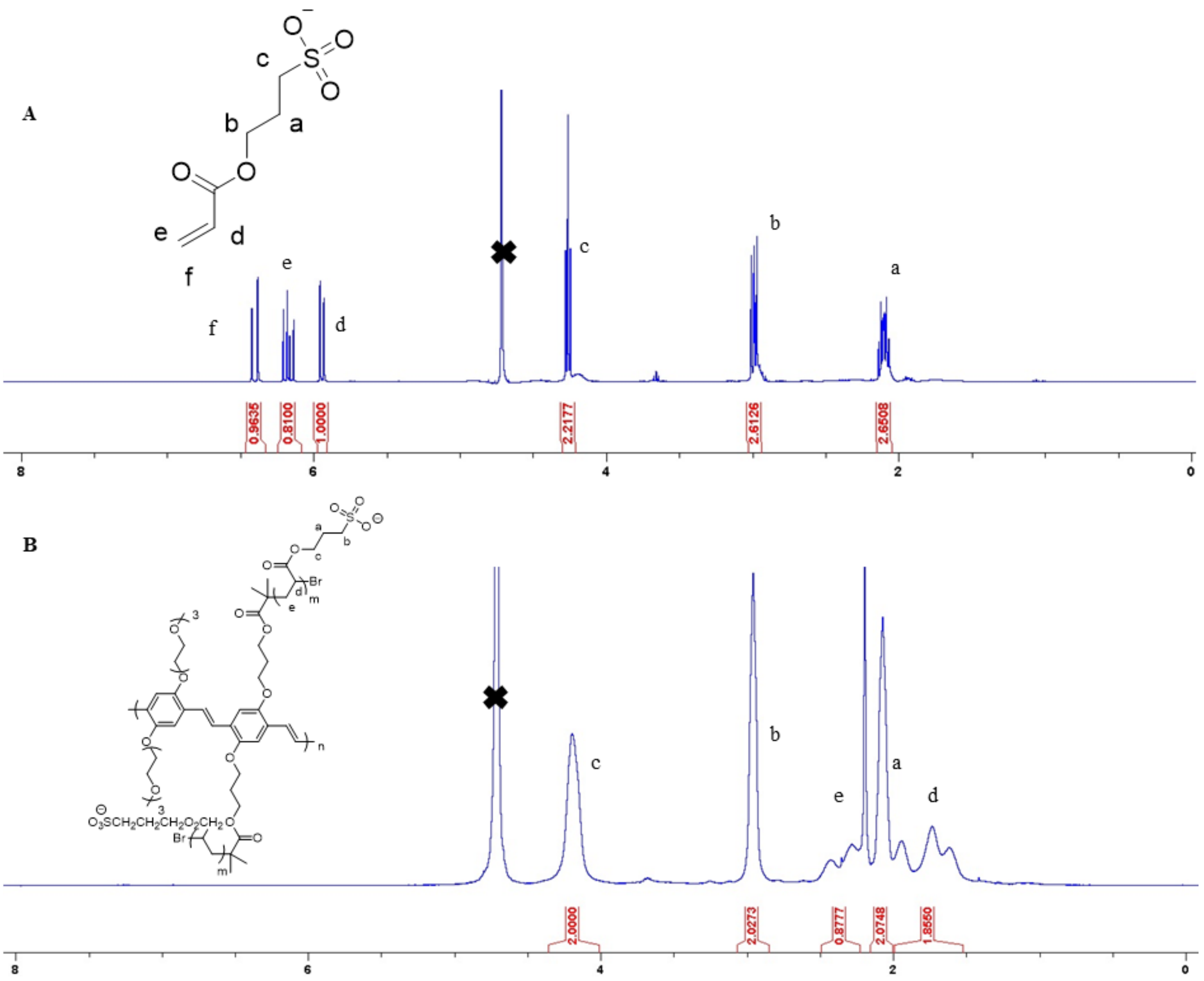

Figure S7. ^{1}H NMR spectra of SPA (A) and PPV-g-PSPA (B) in D_2O. The ^{1}H NMR spectra of PPE-g-PSPA is effectively identical to PPV-g-PSPA due to the high ratio of grafts to backbone.

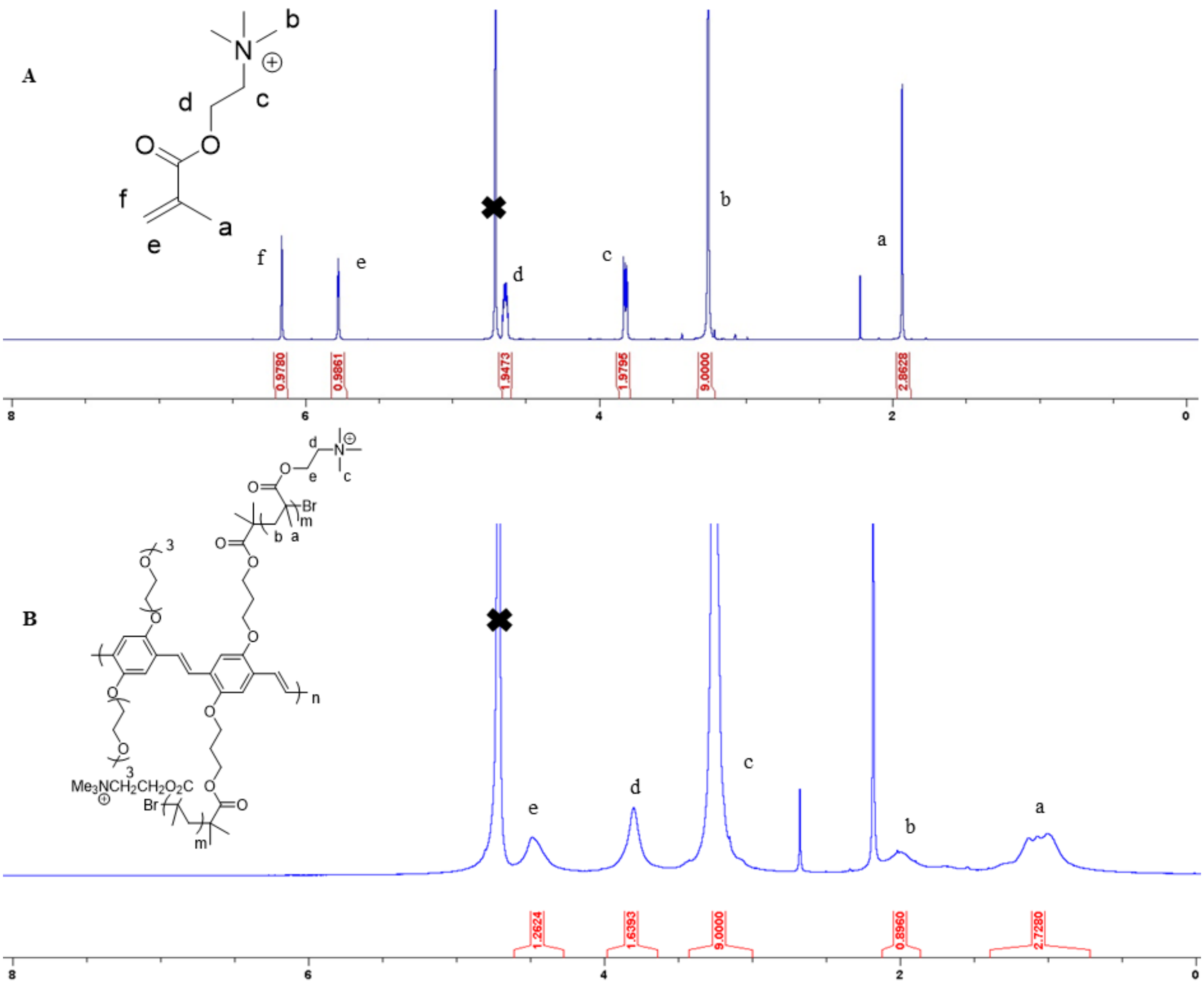

Figure S8. ^{1}H NMR spectra of METAC (A) and PPV-g-PMETAC (B) in D_2O. The ^{1}H NMR spectra of PPE-g-PMETAC is effectively identical to PPV-g-PMETAC due to the high ratio of grafts to backbone.

References

(1) Highly processable, rubbery poly(n-butyl acrylate) grafted poly(phenylene vinylene)s. Baek, P., Kerr-Phillips, T., Damavandi, M., Chaudhary, O. J., Malmstrom, J., Chan, E. W. C., Shaw, P., Burn, P., Barker, D.; Travas-Sejdic, J. *European Polymer Journal* **2016,** *84,* 355.

PPV-g-PSPA Low Molecular Weight

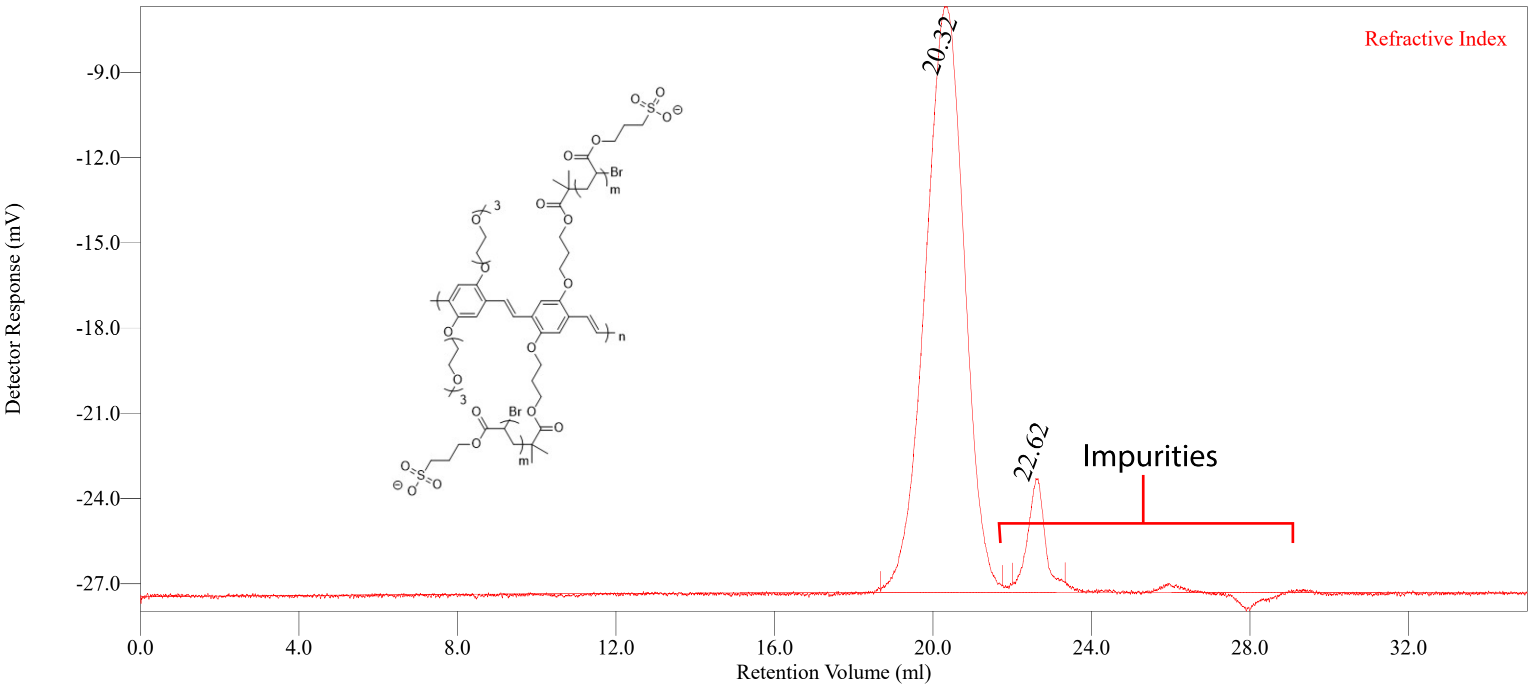

Conventional Calibration - Homopolymers : Results

Peak RV - (ml)	20.317	22.620
Mn - (Daltons)	16,721	1,135
Mw - (Daltons)	22,867	1,237
Mz - (Daltons)	31,861	1,339
Mp - (Daltons)	18,736	1,185
Mw / Mn	1.368	1.090
Percent Above Mw: 0	100.000	100.000
Percent Below Mw: 0	0.000	0.000
Mw 10.0% Low	7,616	667
Mw 10.0% High	55,371	1,968
Wt Fr (Peak)	0.920	0.080
RI Area - (mvml)	23.68	2.05
UV@240nm Area - (mvml)	0.00	0.00

Annotation	
Method File	Dextran in water-0011.vcm
Limits File	
Date Acquired	Feb 28, 2015 - 18:40:22
Solvent	Water 0.02% NaN3
Acquisition Operator	admin : Administrator
Calculation Operator	admin : Administrator
Column Set	2 x A5000
System	System 1
Flow Rate - (ml/min)	1.000
Inj Volume - (ul)	100.0
Volume Increment - (ml)	0.00333
Detector Temp. - (deg C)	35.0
Column Temp. - (deg C)	35.0
OmniSEC Build Number	406

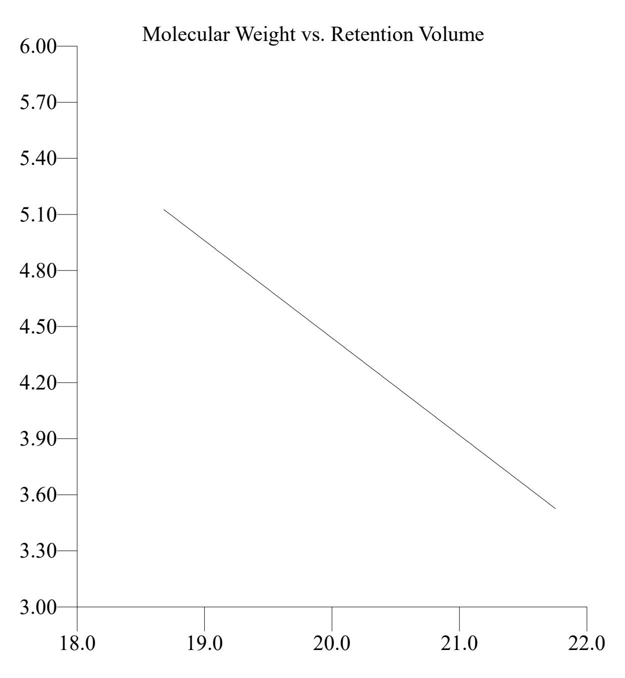

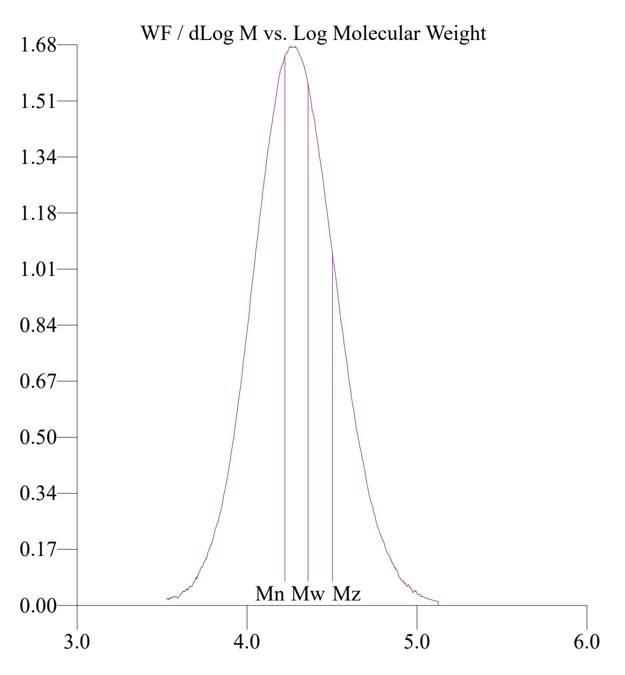

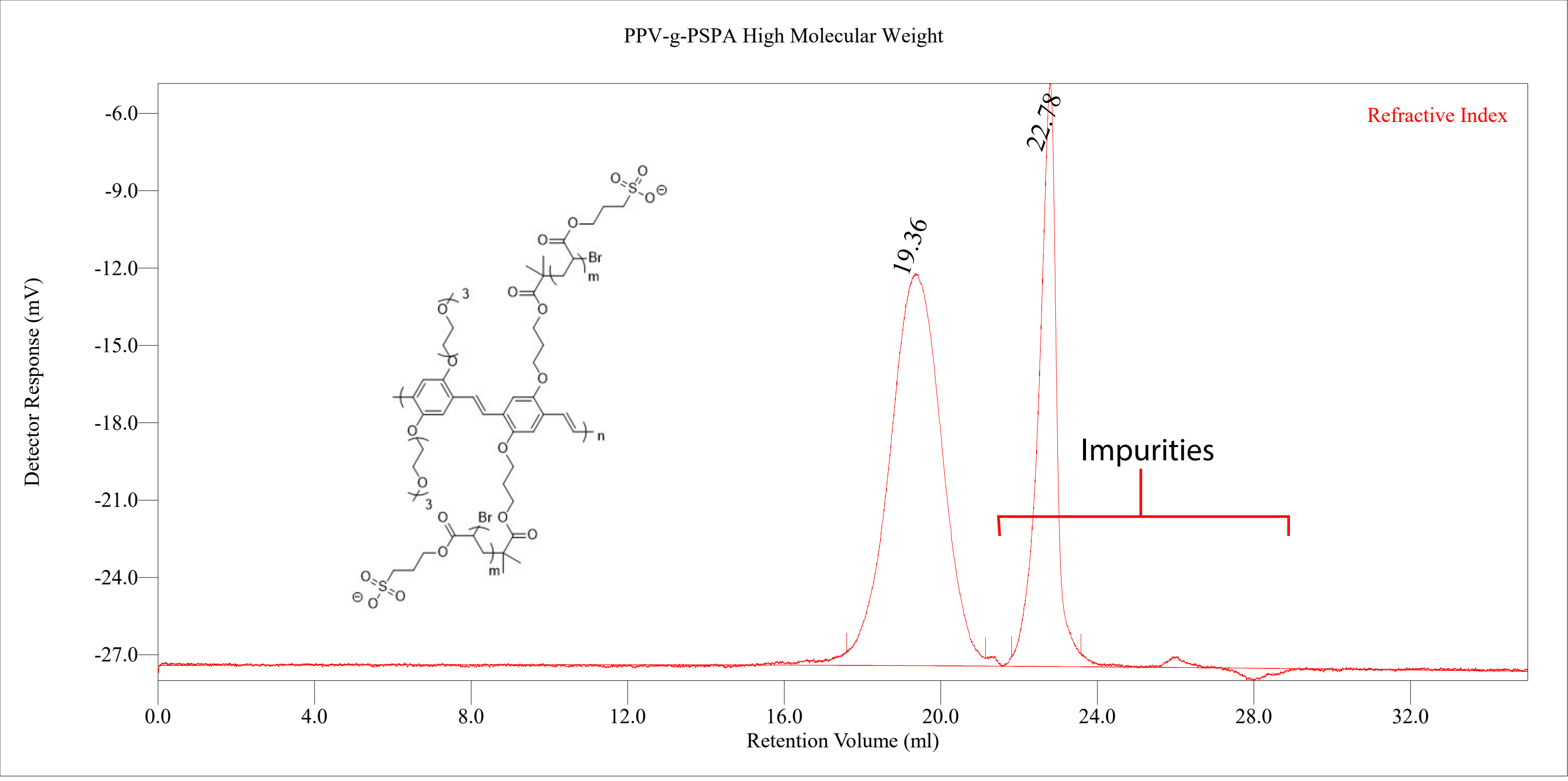

Conventional Calibration - Homopolymers : Results

Peak RV - (ml)	19.363	22.783
Mn - (Daltons)	45,889	1,028
Mw - (Daltons)	78,079	1,139
Mz - (Daltons)	131,586	1,273
Mp - (Daltons)	58,981	973
Mw / Mn	1.701	1.107
Percent Above Mw: 0	100.000	100.000
Percent Below Mw: 0	0.000	0.000
Mw 10.0% Low	17,335	637
Mw 10.0% High	230,745	2,020
Wt Fr (Peak)	0.663	0.337
RI Area - (mvml)	23.34	11.84
UV@240nm Area - (mvml)	0.00	0.00

Annotation	
Method File	Dextran in water-0011.vcm
Limits File	
Date Acquired	Feb 28, 2015 - 22:15:51
Solvent	Water 0.02% NaN3
Acquisition Operator	admin : Administrator
Calculation Operator	admin : Administrator
Column Set	2 x A5000
System	System 1
Flow Rate - (ml/min)	1.000
Inj Volume - (ul)	100.0
Volume Increment - (ml)	0.00333
Detector Temp. - (deg C)	35.0
Column Temp. - (deg C)	35.0
OmniSEC Build Number	406

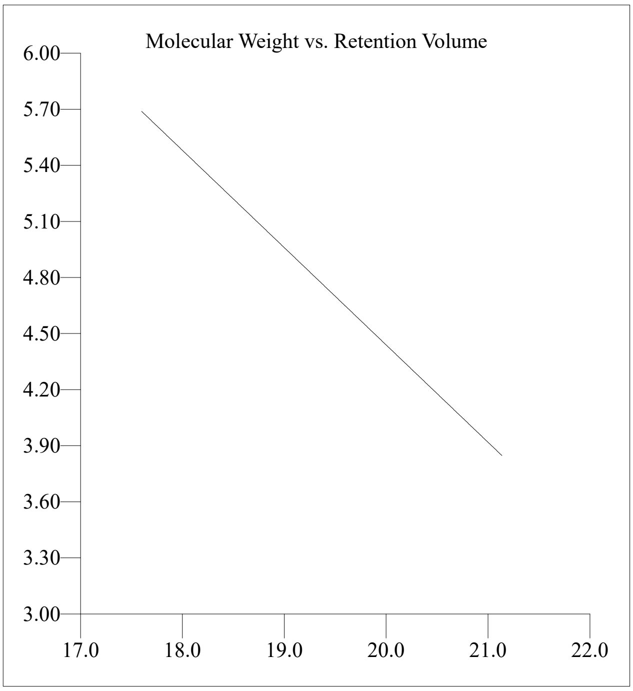

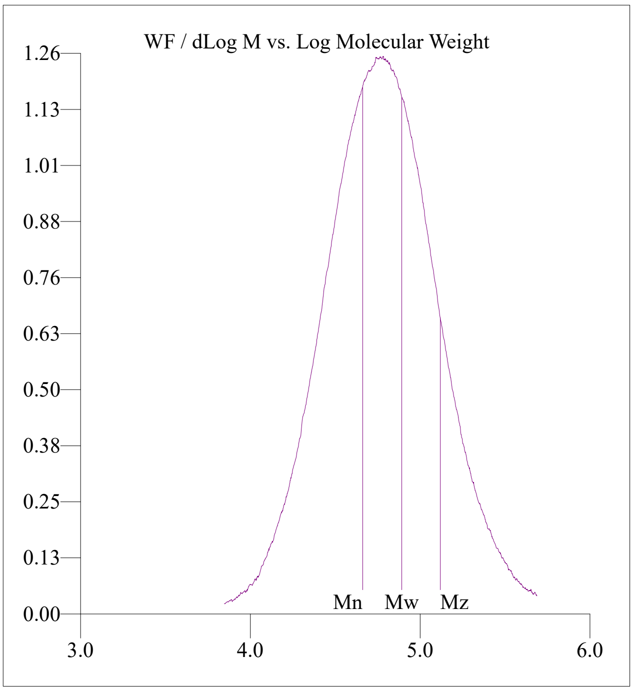

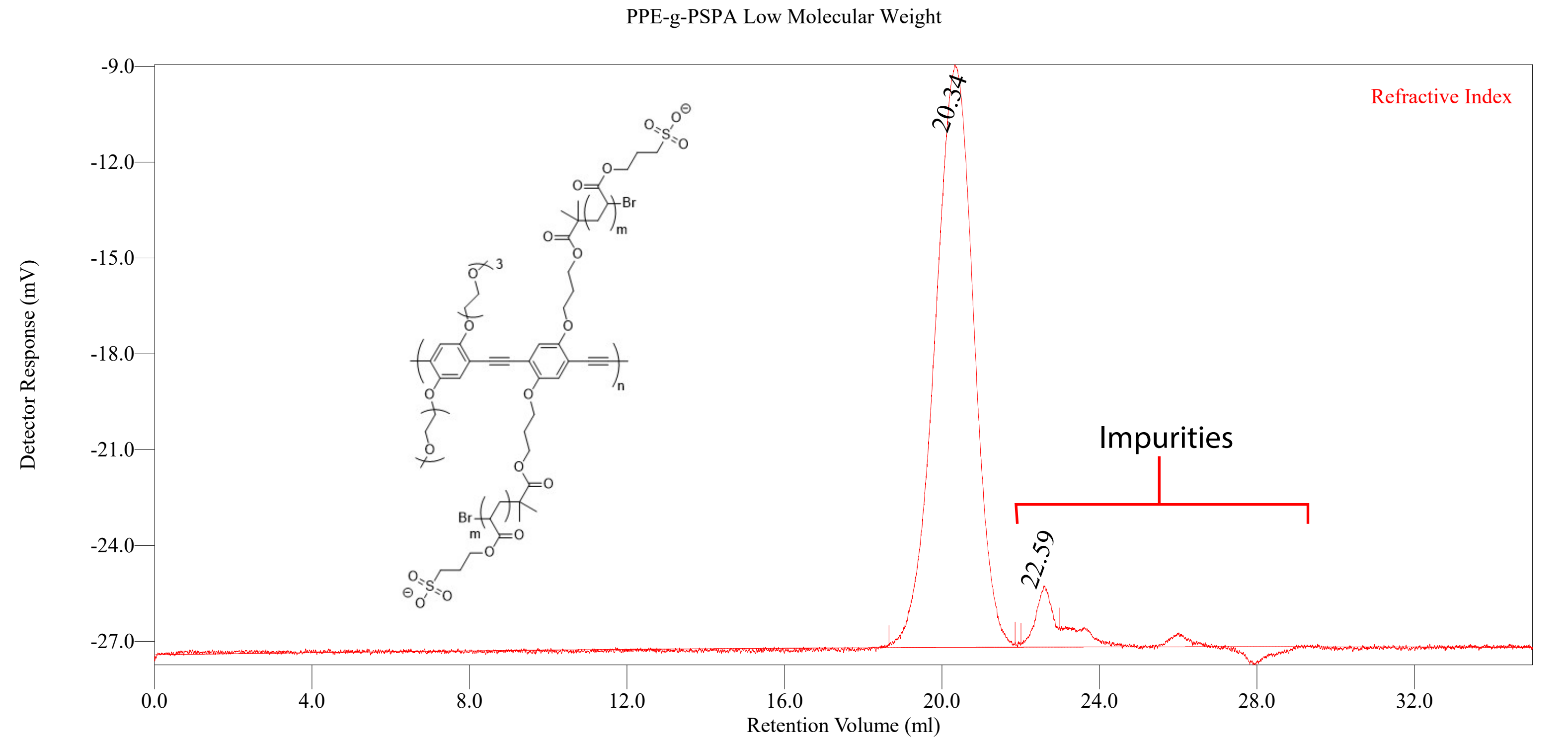

Conventional Calibration - Homopolymers : Results

Peak RV - (ml)	20.337	22.593
Mn - (Daltons)	16,385	1,208
Mw - (Daltons)	22,434	1,281
Mz - (Daltons)	31,320	1,363
Mp - (Daltons)	18,325	1,222
Mw / Mn	1.369	1.060
Percent Above Mw: 0	100.000	100.000
Percent Below Mw: 0	0.000	0.000
Mw 10.0% Low	7,438	834
Mw 10.0% High	54,556	1,968
Wt Fr (Peak)	0.957	0.043
RI Area - (mvml)	20.86	0.93
UV@240nm Area - (mvml)	0.00	0.00

Annotation	
Method File	Dextran in water-0011.vcm
Limits File	
Date Acquired	Feb 28, 2015 - 19:16:18
Solvent	Water 0.02% NaN3
Acquisition Operator	admin : Administrator
Calculation Operator	admin : Administrator
Column Set	2 x A5000
System	System 1
Flow Rate - (ml/min)	1.000
Inj Volume - (ul)	100.0
Volume Increment - (ml)	0.00333
Detector Temp. - (deg C)	35.0
Column Temp. - (deg C)	35.0
OmniSEC Build Number	406

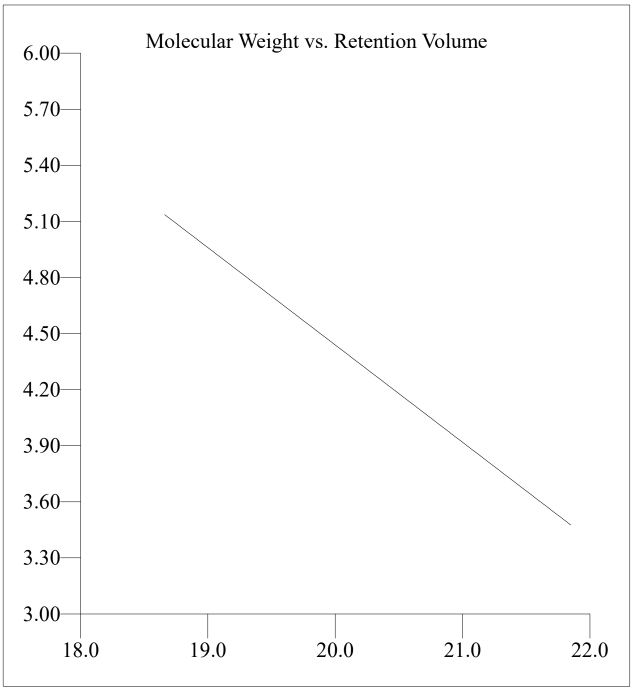

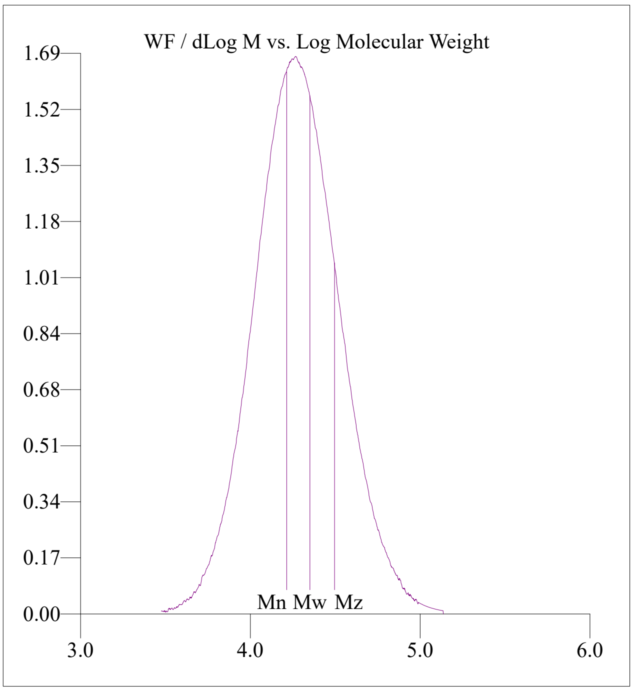

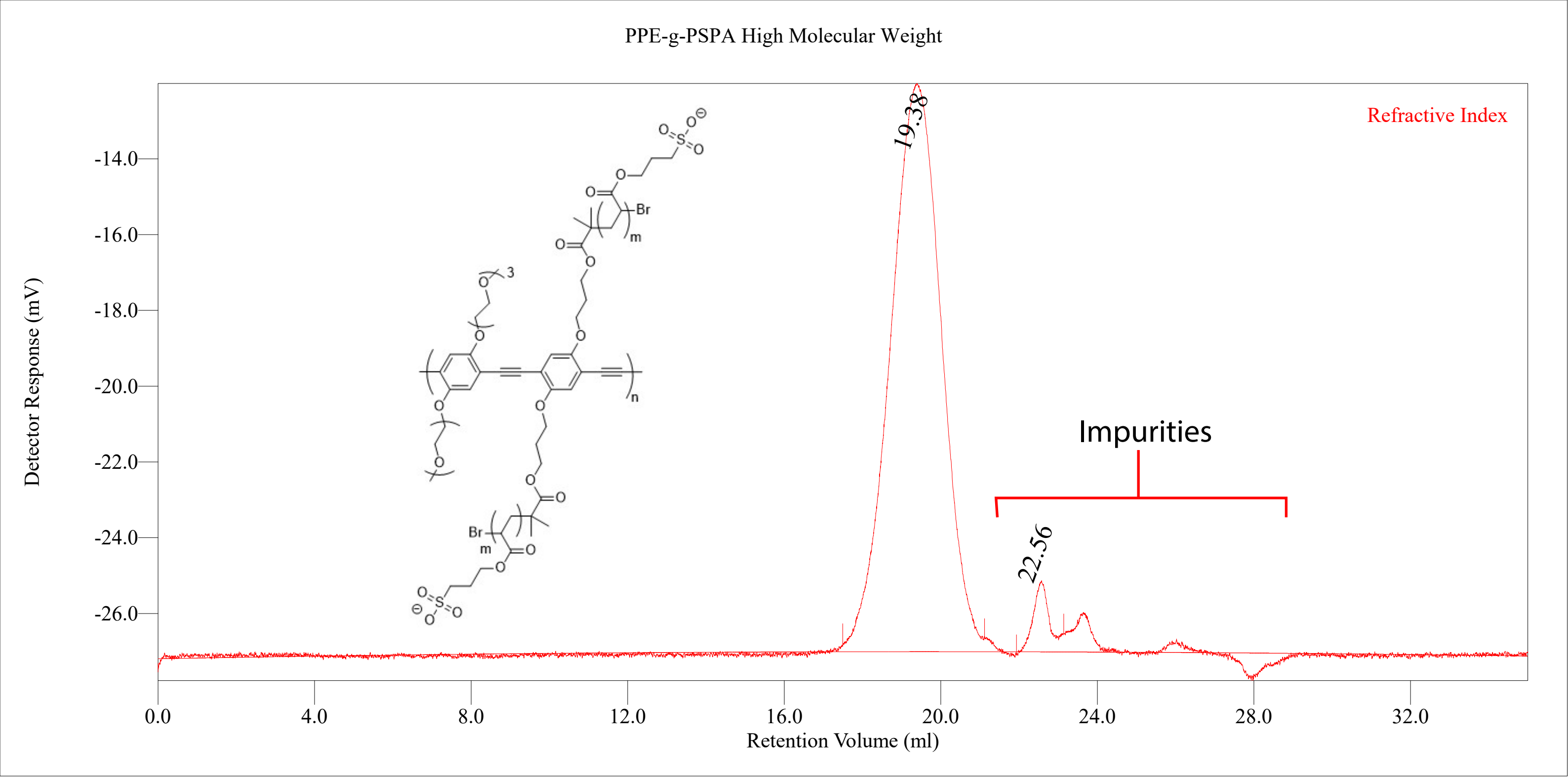

Conventional Calibration - Homopolymers : Results

Peak RV - (ml)	19.383	22.560
Mn - (Daltons)	46,027	1,205
Mw - (Daltons)	77,483	1,311
Mz - (Daltons)	129,822	1,425
Mp - (Daltons)	57,365	1,271
Mw / Mn	1.683	1.088
Percent Above Mw: 0	100.000	100.000
Percent Below Mw: 0	0.000	0.000
Mw 10.0% Low	17,573	722
Mw 10.0% High	226,142	1,865
Wt Fr (Peak)	0.962	0.038
RI Area - (mvml)	22.71	0.89
UV@240nm Area - (mvml)	0.00	0.00

Annotation	
Method File	Dextran in water-0011.vcm
Limits File	
Date Acquired	Feb 28, 2015 - 20:28:08
Solvent	Water 0.02% NaN3
Acquisition Operator	admin : Administrator
Calculation Operator	admin : Administrator
Column Set	2 x A5000
System	System 1
Flow Rate - (ml/min)	1.000
Inj Volume - (ul)	100.0
Volume Increment - (ml)	0.00333
Detector Temp. - (deg C)	35.0
Column Temp. - (deg C)	35.0
OmniSEC Build Number	406

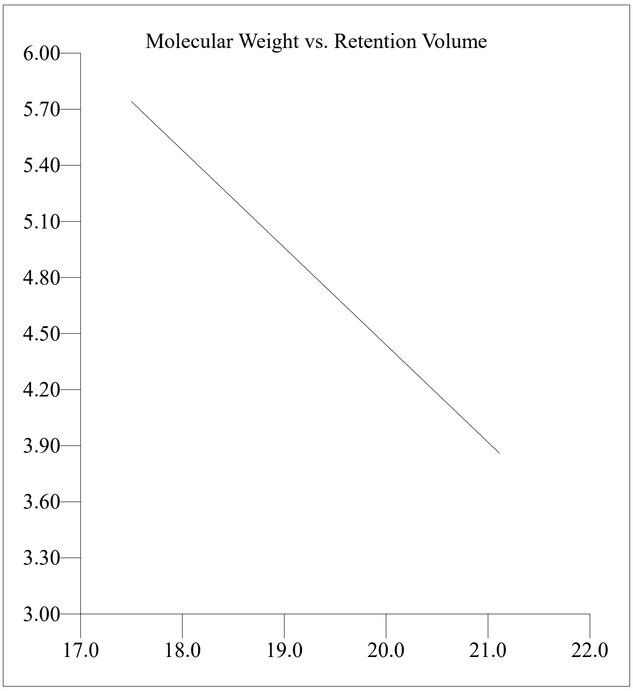

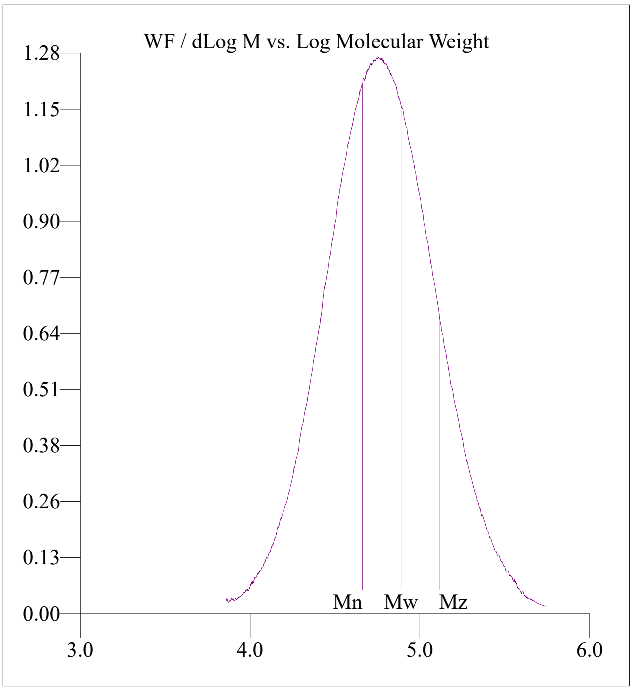

PPV-g-PMETAC High Molecular Weight

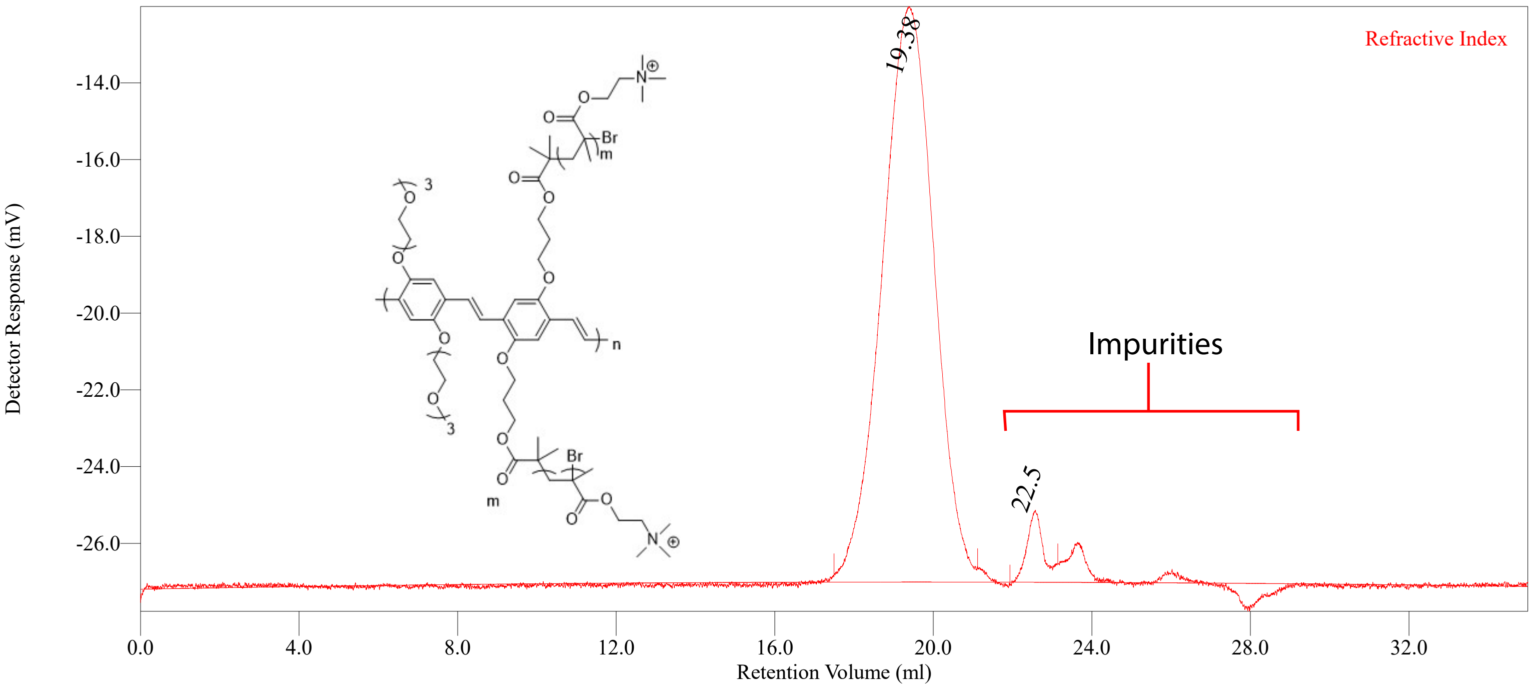

Conventional Calibration - Homopolymers : Results

Peak RV - (ml)	19.383	22.560
Mn - (Daltons)	46,027	1,205
Mw - (Daltons)	77,483	1,311
Mz - (Daltons)	129,822	1,425
Mp - (Daltons)	57,365	1,271
Mw / Mn	1.683	1.088
Percent Above Mw: 0	100.000	100.000
Percent Below Mw: 0	0.000	0.000
Mw 10.0% Low	17,573	722
Mw 10.0% High	226,142	1,865
Wt Fr (Peak)	0.962	0.038
RI Area - (mvml)	22.71	0.89
UV@240nm Area - (mvml)	0.00	0.00

Annotation	
Method File	Dextran in water-0011.vcm
Limits File	
Date Acquired	Feb 28, 2015 - 20:28:08
Solvent	Water 0.02% NaN3
Acquisition Operator	admin : Administrator
Calculation Operator	admin : Administrator
Column Set	2 x A5000
System	System 1
Flow Rate - (ml/min)	1.000
Inj Volume - (ul)	100.0
Volume Increment - (ml)	0.00333
Detector Temp. - (deg C)	35.0
Column Temp. - (deg C)	35.0
OmniSEC Build Number	406

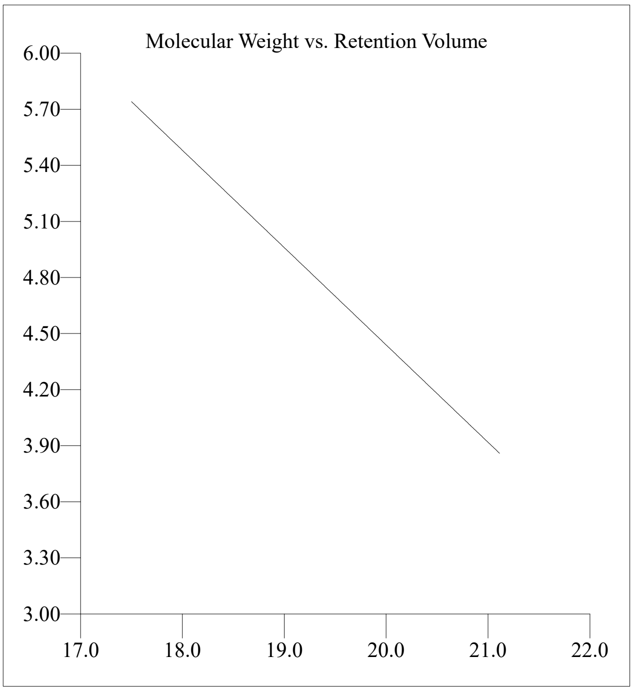

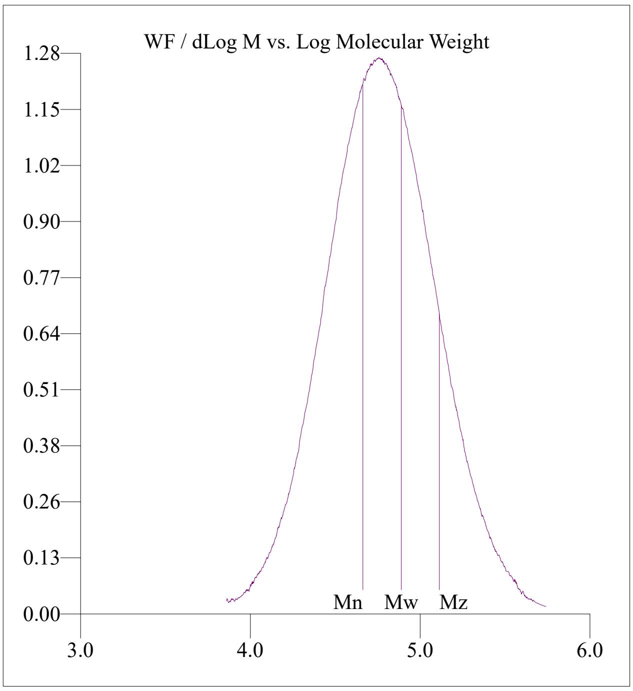

Supplement: Supplementary file 1 [file polymers-14-02767-s001.zip › polymers-1798403-Supplementary.pdf]
